# Supplementary material for: Deep-water circulation changes lead North Atlantic climate during deglaciation
Source: Nat Commun. 2019 Mar 20;10:1272. doi: 10.1038/s41467-019-09237-3 (PMC6426850; doi:10.1038/s41467-019-09237-3)
Supplement: Supplementary file 1 — Supplementary Information [file 41467_2019_9237_MOESM1_ESM.pdf]

Supplementary Information

Deep-water circulation changes lead North Atlantic climate during deglaciation

Francesco Muschitiello et al.

Supplementary Figures

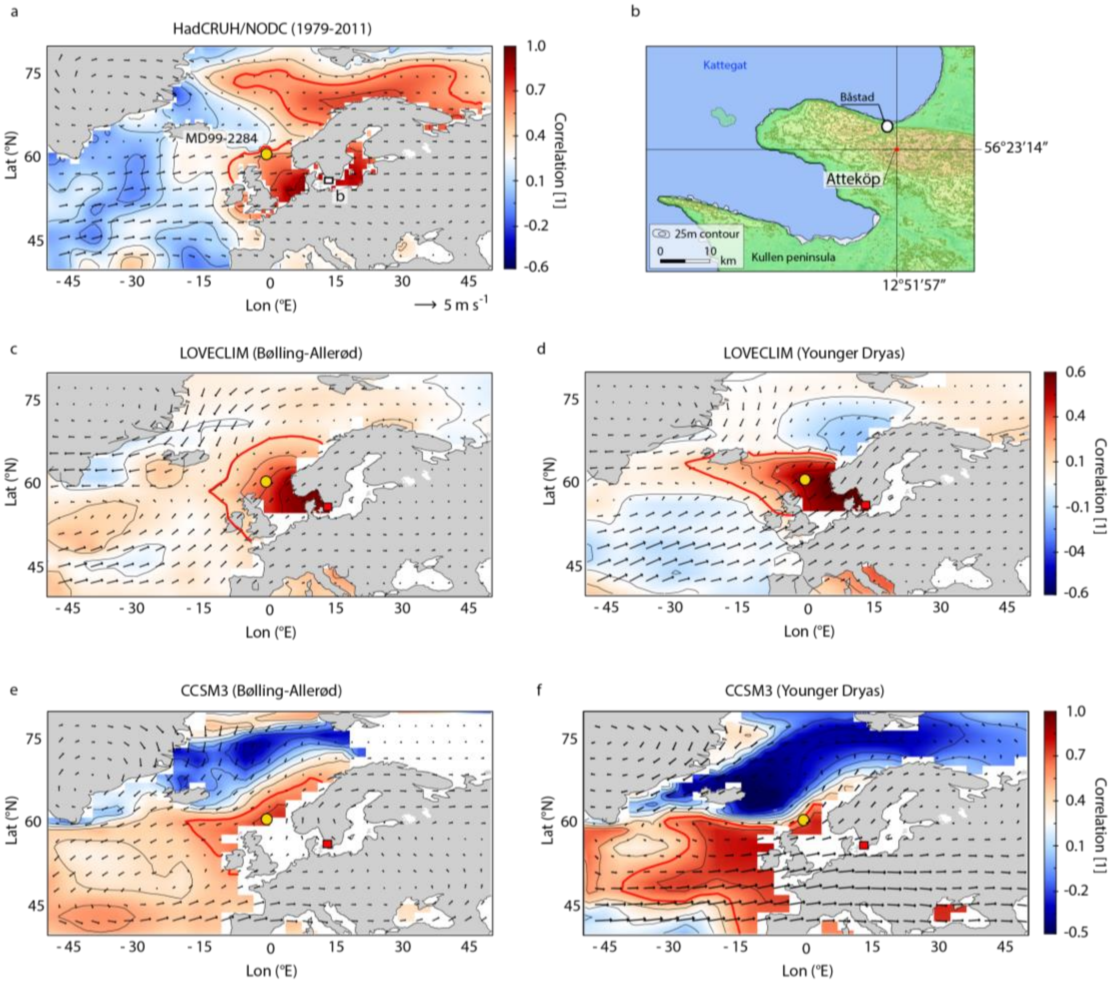

Supplementary Figure 1 – Modern and past sensitivity of hydro-climate in southern Sweden to regional oceanography. a, Climatological field correlation between annual HadCRUH specific humidity [1] in southern Sweden (56-57° N, 12-13° E) and annual NODC 0-100 m vertical mean temperature [2] in the Nordic Seas, highlighting the relationship between water vapour-mass transport to southern Sweden and upwind changes in near-surface ocean temperature. Results are plotted together with annual climatology of NCEP/NCAR 850 hPa wind field [3]. The thick red line defines the area where the correlation is significant above the 95% confidence level when tested against red noise from an AR1 process. All data were detrended before the correlation was estimated. Inset white rectangle represents the area shown in (b). **b**, Digital elevation model of the southwestern coast of Sweden showing the location of lake core site at Atteköp. **c-d**, Same as in (a) for Bølling-Allerød and Younger Dryas 200-year long climatology, respectively, using transient climate simulation results from the coupled atmosphere-ocean-sea ice-carbon cycle model LOVECLIM [4]. **e-f**, Same as in (c-d) but using transient climate simulation results from the coupled atmosphere-ocean-sea ice-land surface climate model CCSM3 [5]. Climate model field correlations were estimated using vertical mean temperatures integrated over the interval 0-250 m. The locations of core MD99-2284 (yellow circle) and Attekøp (red square) are also shown.

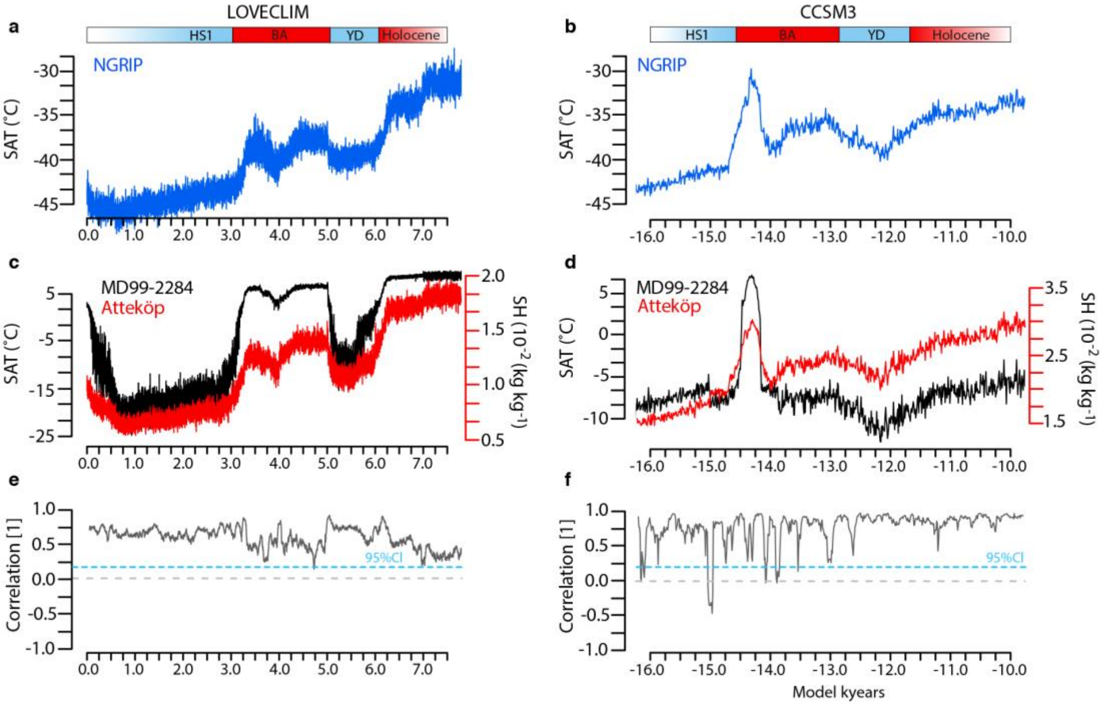

Supplementary Figure 2 – Modelled climate changes during the last deglaciation. a, Surface air temperature (SAT) at NGRIP coring site using transient climate simulation results from LOVECLIM (ref. [4]). **b**, Same as in (a) but using transient climate simulation results from CCSM3 (ref. [5]). **c,** SAT and specific humidity (SH) at site MD99-2284 and at Atteköp, respectively, as simulated using LOVECLIM. **d**, Same as in (c) but using CCSM3. **e**, Correlation (over sliding windows of 100 years) between SAT at MD99-2284 and SH at Atteköp as simulated using LOVECLIM. **f**, Same as in (e) but using CCSM3. The dashed cyan line defines the 95% confidence level inferred via correlation of the SH series to 1,000 synthetic series with similar AR1 characteristics to the SAT data. Model output are presented on their independent time scale. Notable climatic transitions are shown at the top. HS1: Heinrich Stadial 1; BA: Bølling-Allerød Interstadial; YD: Younger Dryas Stadial.

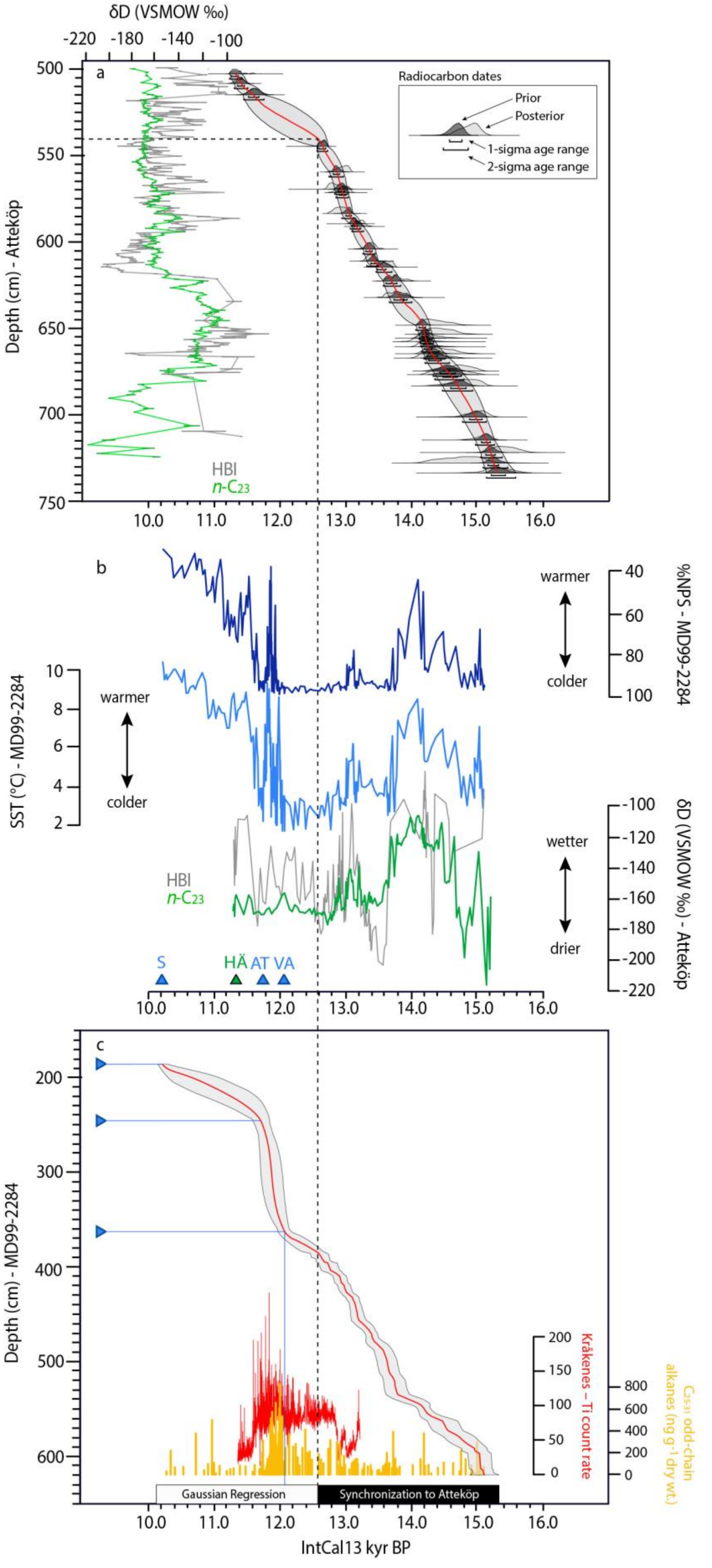

δD (VSMOW ‰)
a
Radiocarbon dates
Prior
Posterior
1-sigma age range
2-sigma age range
Depth (cm) - Atteköp
HBI
n-C23
b
warmer
colder
%NPS - MD99-2284
SST (°C) - MD99-2284
wetter
drier
δD (VSMOW ‰) - Atteköp
S
HÄ
AT
VA
c
Depth (cm) - MD99-2284
Kråkenes – Ti count rate
C25-31 odd-chain alkanes (ng g-1 dry wt.)
Gaussian Regression
Synchronization to Atteköp
IntCal13 kyr BP

Supplementary Figure 3 – Age model construction. **a**, Hydro-climate record from Atteköp lake sediments based on leaf-wax δD measurements obtained from aquatic macrophyte-sourced C_{23}-alkanes (green) and diatom-sourced C_{20} highly-branched isoprenoids (HBIs, grey) plotted versus depth, and Bayesian age-depth model for Atteköp [6]. The δD record is presented with 2σ error bars associated with triplicate δD measurements. **b**, Synchronized sea-surface temperature (SST) record from core MD99-2284 (light blue) and δD record from Atteköp lake sediments using an automated Monte Carlo alignment algorithm [7,8]. The relative abundance of downcore *Neogloboquadrina pachyderma* (*s*) (blue) from MD99-2284 is also shown for comparison. The triangles indicate the position of the tephra horizons identified in core MD99-2284 (S: Saksunarvatn; AT: Abernethy tephra; VA: Vedde Ash) (blue) and at Atteköp (HÄ: Hässeldalen) (green). Note that the alignment between the marine and terrestrial proxy records was performed only prior to 12500 years BP where the chronology of Atteköp is well constrained. Upcore, the chronology of MD99-2284 was established using the available tephra markers. **c**, Inferred age-depth relationship for core MD99-2284 based on alignment to Atteköp δD record (lower portion) and by fitting the tephra-based age constraints using a Gaussian Regression model [9] (upper portion). Envelopes reflect the 95% confidence level of the age model whereas the red line indicates the median age-depth function. Abundance of long-chain terrestrial *n*-alkanes in core MD99-2284 and glacier mass turnover reconstruction from Lake Kråkenes [10], western Norway, (on the IntCal13 time scale) are also presented. The Vedde Ash has also been identified in Lake Kråkenes and the tephra isochron is displayed (blue line). Note the rapid increase in sedimentation rates around 12000 years BP concomitant with higher input of terrigenous material and higher mass turnover rates of western Norwegian glaciers.

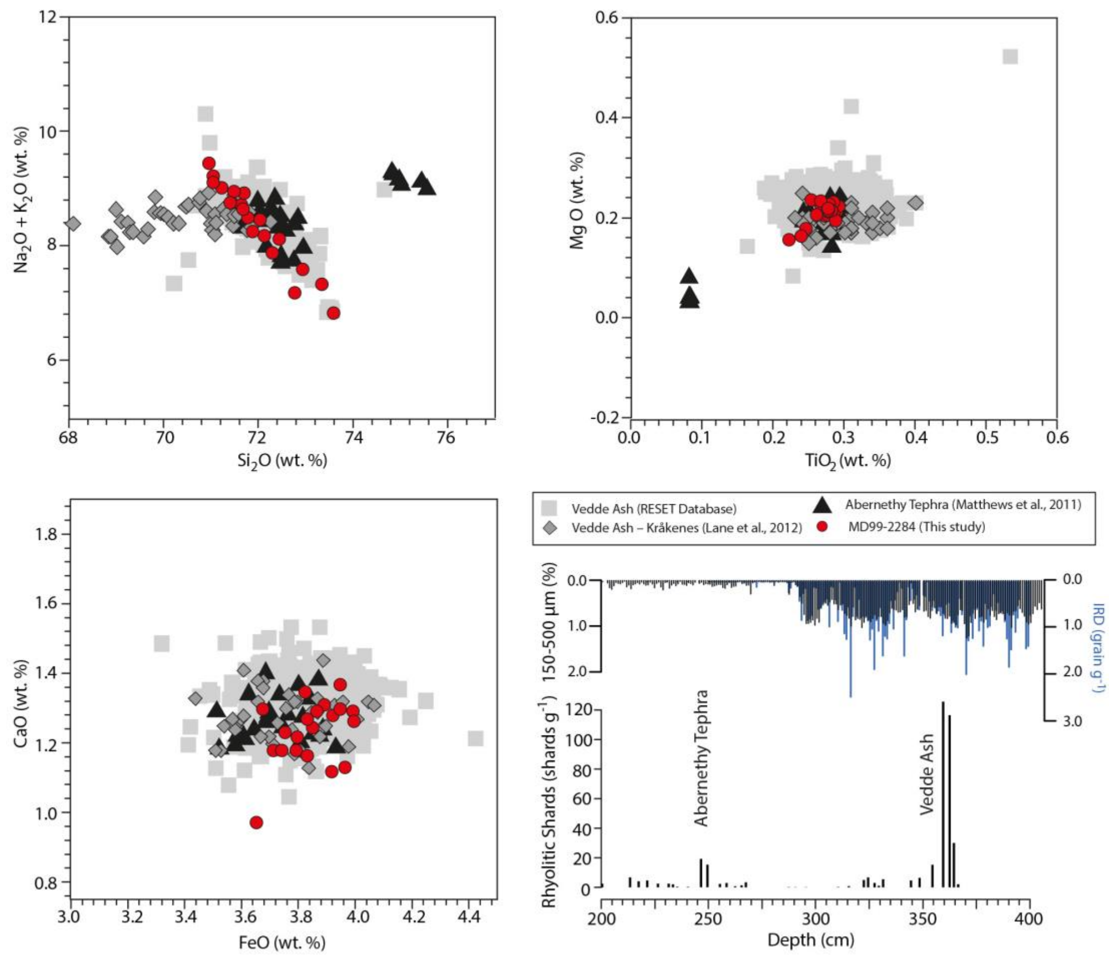

Supplementary Figure 4 – Chemical characteristics of the Abernethy Tephra. Normalized tephra geochemical data from core MD99-2284 (247.5-249.5 cm) compared to data for the Vedde Ash from the RESET Database [11] (http://c14.arch.ox.ac.uk/reset/), the Vedde Ash identified at Lake Kråkenes [12], in Norway, and the Abernethy Tephra identified at Abernethy Forest (AF555) [13], in Scotland. MD99-2284 ice-rafted debris, grain size range for very fine to medium sand (150-500 μm), and tephra record highlighting the position of the Abernethy Tephra are also shown.

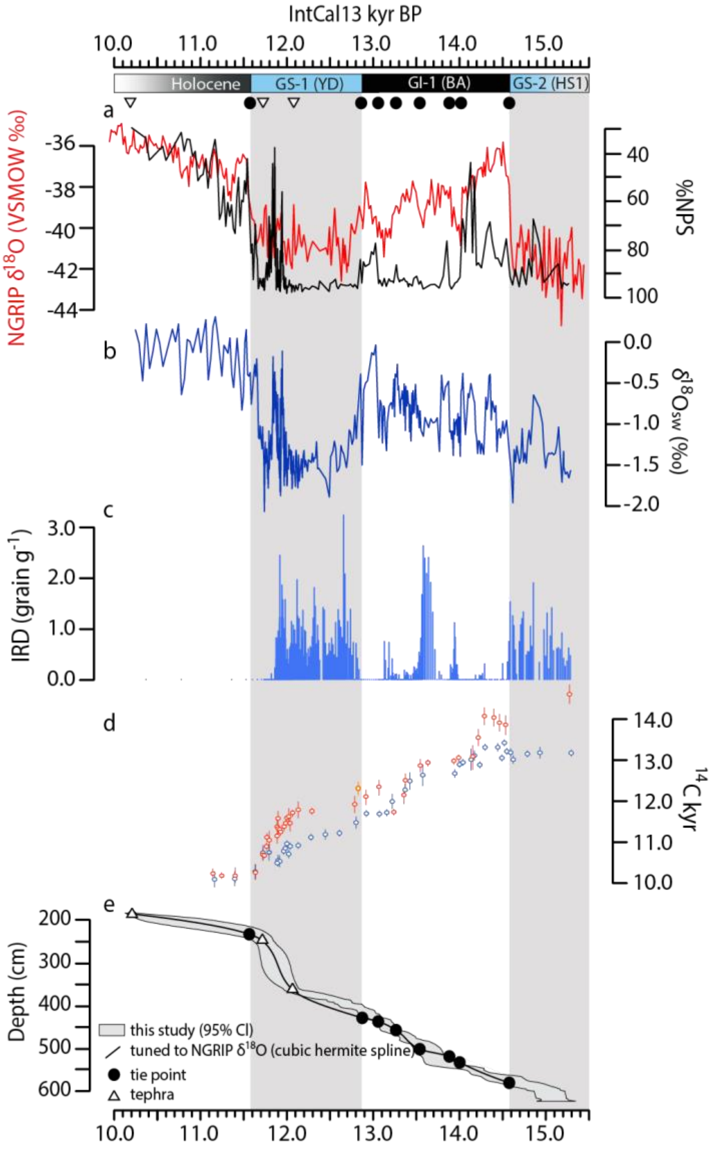

Supplementary Figure 5 – Alternative chronology based on stratigraphic tuning to Greenland ice cores. a, Matching between NGRIP $\delta^{18}O$ record (red) and relative abundance of *Neogloboquadrina pachyderma* (*s*) (NPS - black) from MD99-2284. Tie points and tephra markers used to construct the NGRIP-tuned chronology are presented at the top. Tie points were estimated using a Bayesian change point procedure [14]. **b**, Ice rafted debris count in core MD99-2284 on the NGRIP-tuned time scale. **c**, Raw benthonic (red) and planktic (blue) ^{14}C ages measured in core MD99-2284 and placed on the tuned timescale. Error bars indicate the related 2σ error. Orange dot reflect individual early Younger Dryas ^{14}C measurement from core JM11-FI-19PC [15]. Note that stratigraphically bottom and surface ^{14}C ages diverge shortly before a warming phase preceding GS-1 cooling as observed in the %NPS record. Analogously, bottom and surface ^{14}C ages converge during a phase of cooling preceding the Holocene warming as recorded along the %NPS stratigraphy. **d**, Comparison between the Greenland tuned age-depth model and the chronology used in this study and presented in Supplementary Figure 3. Greenland stratigraphic events relative to the IntCal13 timescale are displayed at the top (GS: Greenland Stadial; GI: Greenland Interstadial; YD: Younger Dryas Stadial; BA: Bølling-Allerød Interstadial; HS1: Heinrich Stadial 1).

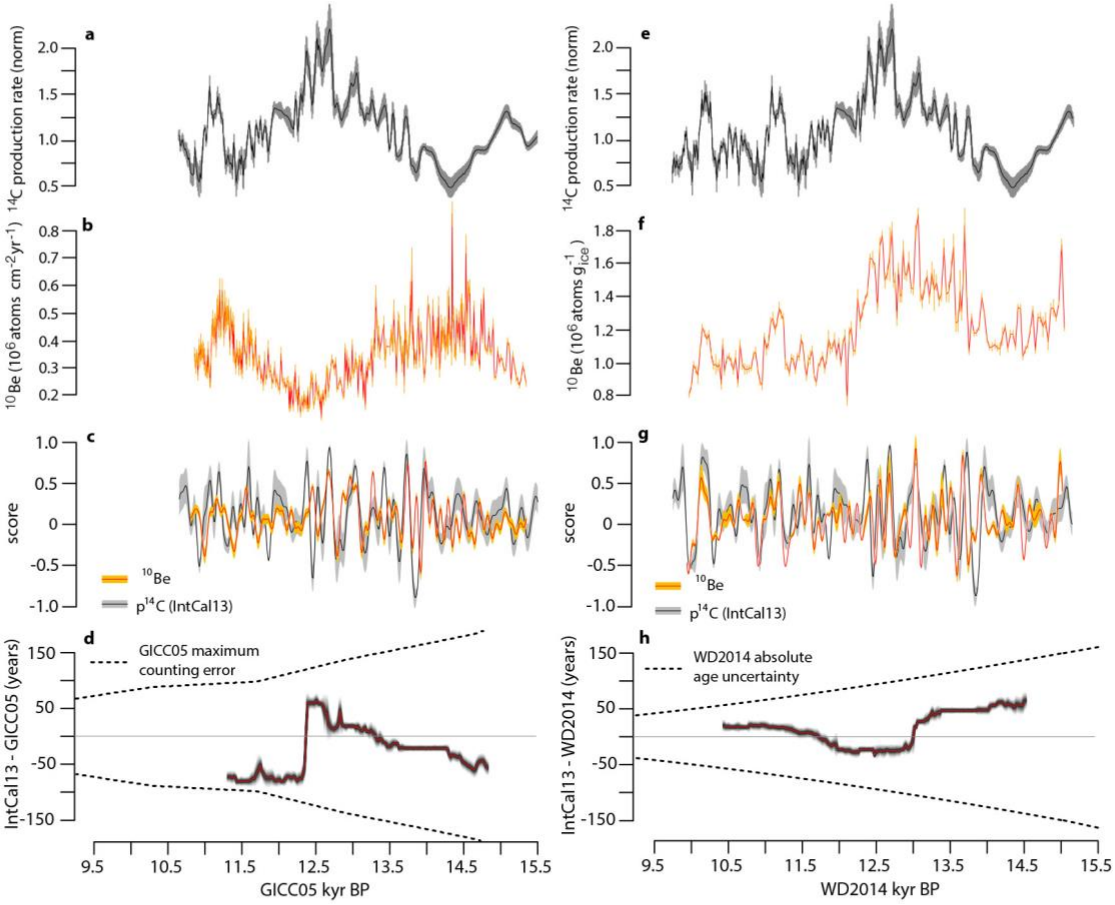

Supplementary Figure 6 – Timescale synchronization. a, Atmospheric ^{14}C production rate inferred from IntCal13 Δ^{14}C. Black line indicates average values and grey envelopes the 2σ error. **b**, GRIP ^{10}Be flux record (red lines) plotted together with errors of individual ^{10}Be measurements (orange bars) [16]. **c**, Comparison of filtered and scaled IntCal13 atmospheric ^{14}C production (black) and GRIP ^{10}Be flux data (red) prior to synchronization. Uncertainty envelopes indicate the 95% confidence level. **d**, Estimated age offset between IntCal13 and GICC05 timescales using a Bayesian wiggle-matching methods (see Methods for details). The red line reflects the most likely age offset and shadings show the 95% confidence level for the estimate. Dashed lines represent the GICC05 maximum counting uncertainty [17]. **e-g**, Same as in (a-c) but using WAIS Divide ^{10}Be concentration data. **h**, Estimated age offset between IntCal13 and WD2014 timescales. Dashed lines represent the WD2014 absolute age uncertainty bounds [18]. The abrupt break in the age offset observed in (d) during the Younger Dryas is consistent with previous results [19] and partly due to a mismatch of tree chronologies forming the IntCal13 data set around to 12500 years BP [19]. A too old placement of the floating tree chronologies that forms IntCal13 before 12000 years BP in conjunction with a systematic undercounting of layers in the ice cores during GS-1 (about 40 uncertain years) [17] may explain the sharp break [19]. In addition, the sparse tree-ring ^{14}C data during this interval make the structure in ^{14}C production rate more uncertain. On the other hand, the break in age offset observed in (h) is likely entirely associated with an erroneous placement of tree-ring data in IntCal13 and a more uncertain structure in the ^{14}C production rate estimates. Note that the GICCO5 b2k convention is here converted to years BP.

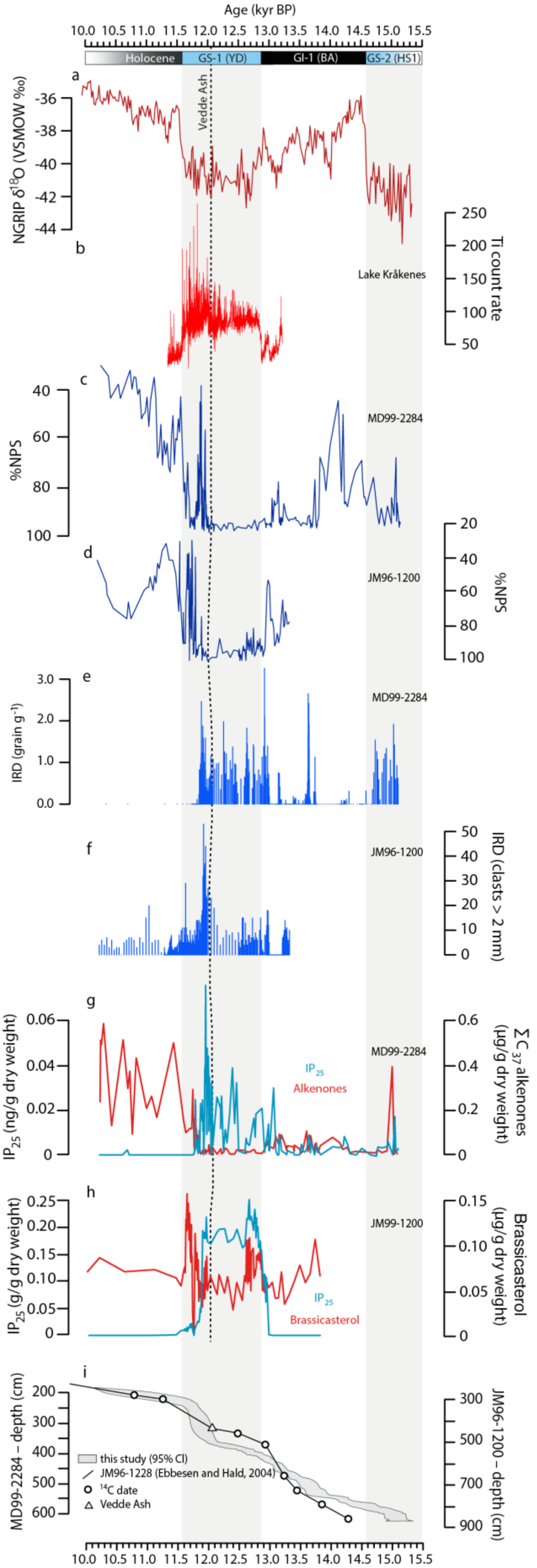

Age (kyr BP)
10.0 10.5 11.0 11.5 12.0 12.5 13.0 13.5 14.0 14.5 15.0 15.5
Holocene
GS-1 (YD)
GI-1 (BA)
GS-2 (HS1)
a
Vedde Ash
NGRIP δ18O (VSMOW ‰)
-36
-38
-40
-42
-44
b
Lake Kråkenes
Ti count rate
250
200
150
100
50
c
MD99-2284
%NPS
40
60
80
100
d
JM96-1200
%NPS
20
40
60
80
100
e
MD99-2284
IRD (grain g-1)
3.0
2.0
1.0
0.0
f
JM96-1200
IRD (clasts > 2 mm)
50
40
30
20
10
0
g
MD99-2284
IP25 (ng/g dry weight)
0.06
0.04
0.02
0
IP25
Alkenones
Σ C37 alkenones (µg/g dry weight)
0.6
0.4
0.2
0
h
JM99-1200
IP25 (g/g dry weight)
0.25
0.20
0.15
0.10
0.05
0
IP25
Brassicasterol
Brassicasterol (µg/g dry weight)
0.15
0.10
0.05
0
i
MD99-2284 – depth (cm)
200
300
400
500
600
this study (95% CI)
JM96-1228 (Ebbesen and Hald, 2004)
14C date
Vedde Ash
JM96-1200 – depth (cm)
300
500
700
900
10.0 10.5 11.0 11.5 12.0 12.5 13.0 13.5 14.0 14.5 15.0 15.5

Supplementary Figure 7 – Comparison with high-resolution records from the northern Norwegian Sea. a, Greenland stratigraphic events and NGRIP $\delta^{18}O$ data on the IntCal13 time scale. **b**, Glacier mass turnover reconstruction from Lake Kråkenes [10], western Norway, on the IntCal13 time scale. **c-d**, Relative abundance of *Neogloboquadrina pachyderma* (*s*) in core MD99-2284 (this study) and JM96-1200 (ref. [20]). Note that the rapid warming at the onset of GI-1 in Greenland is within the chronological uncertainty of the surface warming observed in core MD99-2284. **e-f**, Ice-rafted debris count in core MD99-2284 and JM96-1200. **g,** Concentration of sea-ice diatom biomarker IP_{25} and phytoplankton biomarkers C_{37} unsaturated alkenones in core MD99-2284. **h**, Concentration of sea-ice diatom biomarker IP_{25} and phytoplankton biomarker brassicasterol in core JM99-1200 (ref. [21]). **i,** Comparison between MD99-2284 age-depth model and JM96-1200 age-depth relationship [20]. Note the increase in sedimentation rates above the Vedde Ash in both cores. Proxy records from core JM99-1200/JM96-1200 were generated at the same coring location and are presented on their independent chronology as published in ref. [20]. Greenland stratigraphic events relative to the IntCal13 timescale are displayed at the top (GS: Greenland Stadial; GI: Greenland Interstadial; YD: Younger Dryas Stadial; BA: Bølling-Allerød Interstadial; HS1: Heinrich Stadial 1).

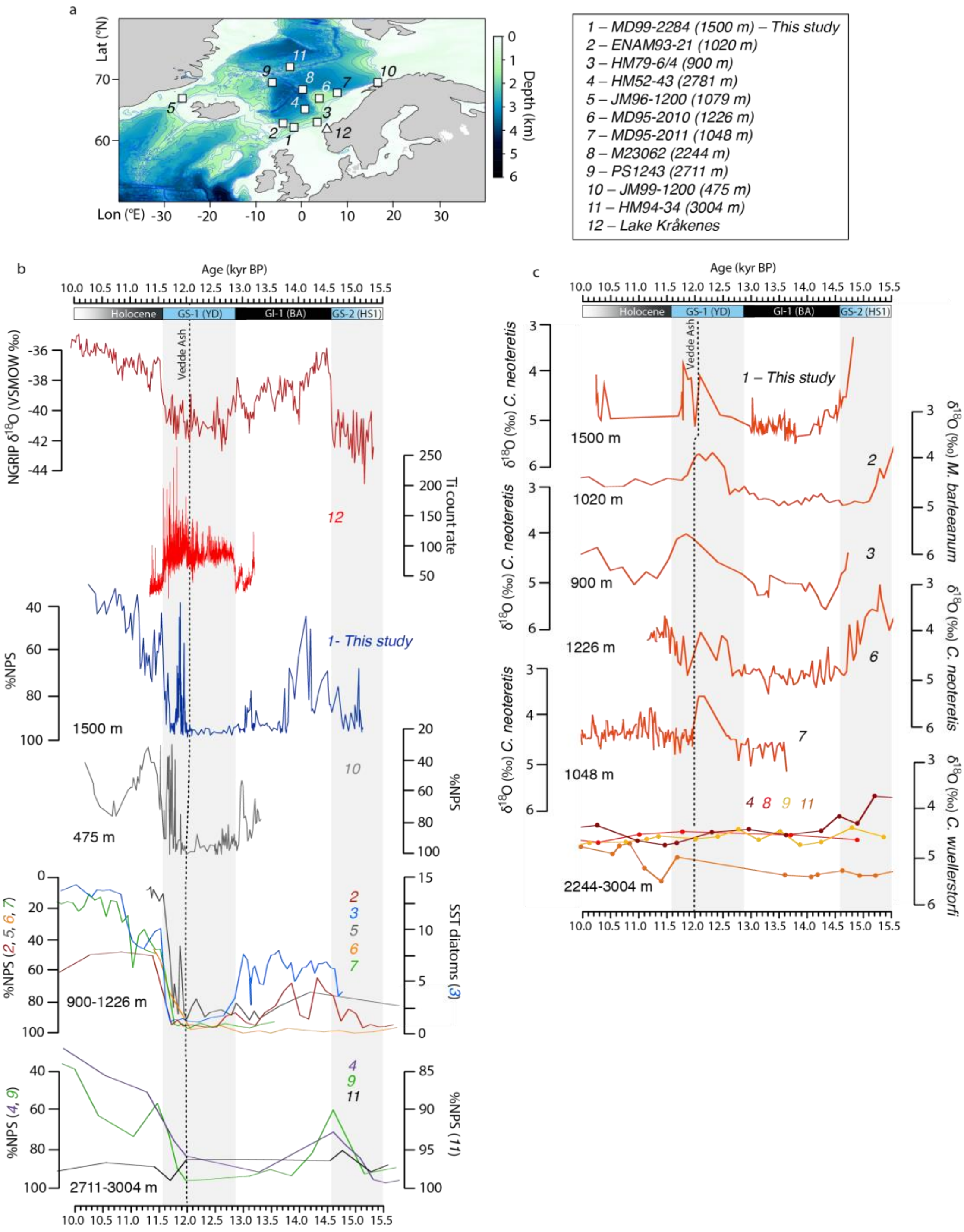

Supplementary Figure 8 – Comparison of pale-oceanographic records from the Nordic Seas during deglaciation. a, Bathymetric map of the Nordic Seas and sediment cores used for inter-site comparisons of sea-surface temperature conditions (%NPS and SST) and bottom water mass properties ($\delta^{18}O$ of benthonic foraminifera – $\delta^{18}O_b$). **b**, Comparison of %NPS data from core MD99-2284 (this study) and other published downcore %NPS and SST records [22], together with Greenland stratigraphic events and NGRIP $\delta^{18}O$ data on the IntCal13 time scale (GS: Greenland Stadial; GI: Greenland Interstadial; YD: Younger Dryas Stadial; BA: Bølling-Allerød Interstadial; HS1: Heinrich Stadial 1). Glacier mass turnover reconstruction from Lake Kråkenes [10], western Norway, on the IntCal13 time scale

is also presented. **c**, Comparison of $\delta^{18}O_b$ data from core MD99-2284 (this study) and other published records from the Norwegian Sea. All published records in (b) and (c) are presented on their independent chronology as constructed in ref. [22]. Note that mismatches between the records can be due to genuine time lags among sites but more likely to inconsistencies among the chronologies, which are based on a combination of four different techniques: *i*) tie points based on the assumption that abrupt warming/cooling in Greenland temperatures and North Atlantic SST are synchronous; *ii*) synchronization to Greenland using the Vedde Ash (dashed line); *iii*) shifts in planktic $\delta^{18}O$ events used to define the end of the LGM and peak melting events; *iv*) calibrated ^{14}C ages using a constant R correction of 400 years.

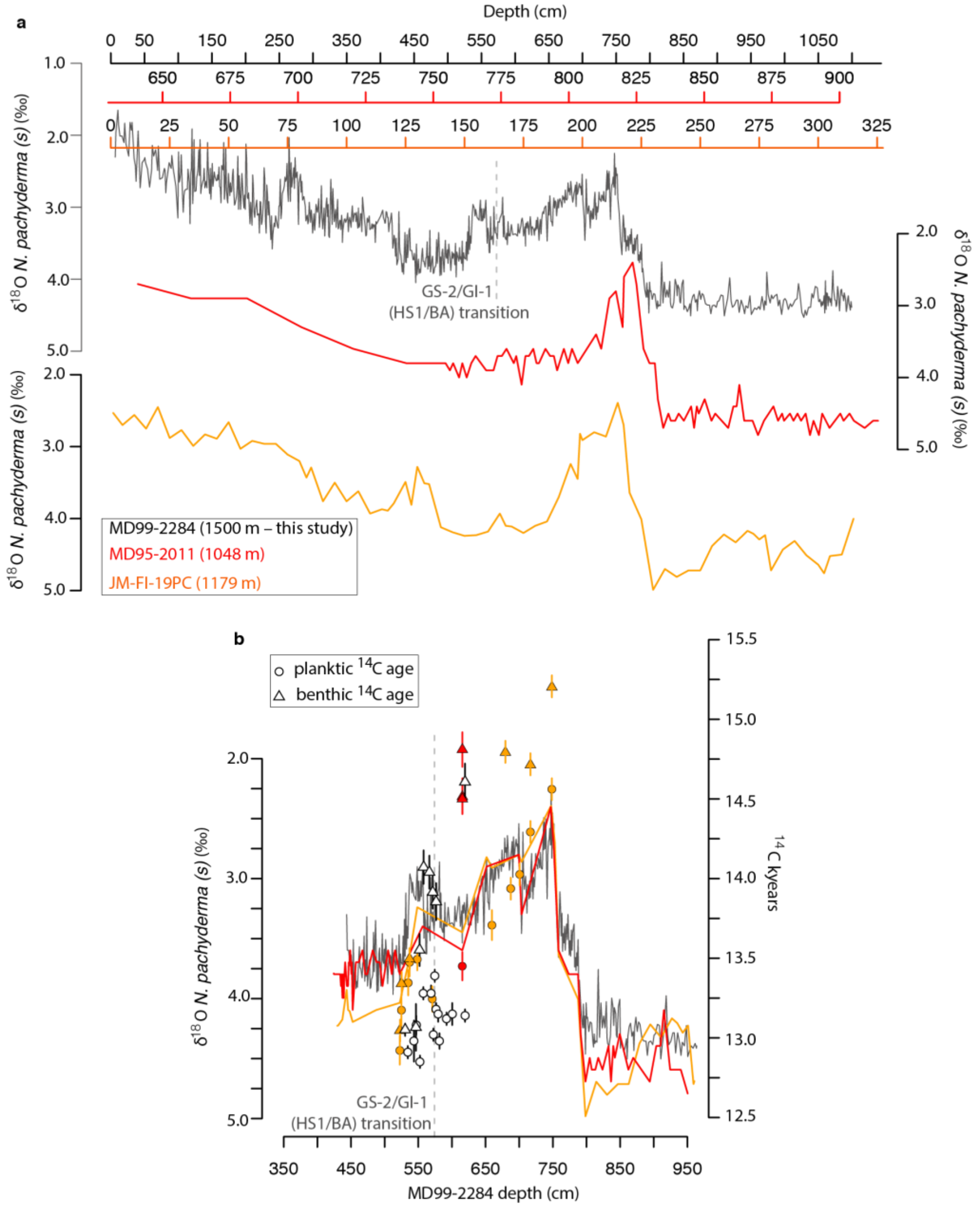

Supplementary Figure 9 – Pre-Greenland Interstadial-1 B-P estimates from the Norwegian Sea. a, Independent planktonic $\delta^{18}O$ records from core MD99-2284, MD95-2011 (Vøring Plateau) [23], and JM-FI-19PC [15] based on *N. pachyderma (s).* Grey dashed line indicates the stratigraphic position of the GS-2/GI-1 (Heinrich Stadial 1/Bølling) boundary in core MD99-2284. **b**, Alignment of core MD95-2011 and JM-FI-19PC to MD99-2284 through correlation of *N. pachyderma (s)* $\delta^{18}O$. Published ^{14}C dates from planktic (circles) and benthic (triangles) material for each core are also shown. The three bottom ^{14}C measurements in core MD95-2011 were derived from one solitary deep-sea coral, whereas the corresponding surface ^{14}C was based on one *N. pachyderma (s)* date. Stratigraphic alignment was obtained using the same Monte Carlo approach we employed to synchronize our terrestrial and marine proxy records (Methods) [7,8,24]. Note that the deep-sea coral in MD95-2011 was absolutely dated using U/Th dating and the proposed alignment presented here, when converted into age, falls within the 95% confidence level of the U/Th age.

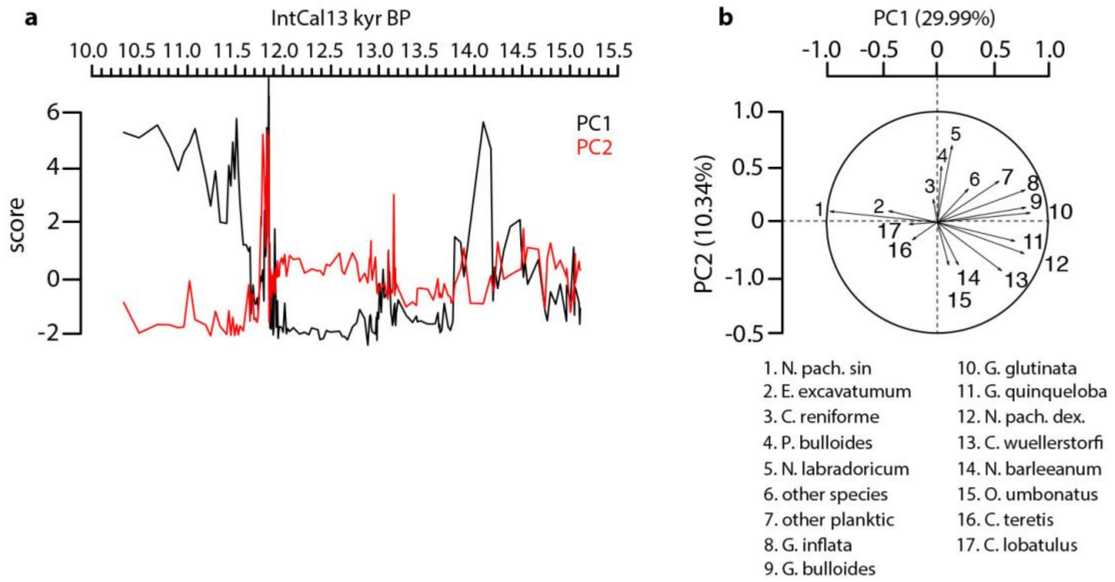

Supplementary Figure 10 – Principal component analysis of foraminifera counts. a, First two principal components of the relative percentages of 17 species of planktonic and benthonic foraminifera reconstructed in core MD99-2284. **b**, Variable factor map showing the correlation coefficients of the variables on the principal components. Percentage of explained variance associated with each component is also shown. The second principal component of all foraminifera counts in core MD99-2284 explains 10.3% of the total variance and is dominated by *N. labradoricum* (correlation coefficient on the PC2 r = 0.69), which is an indicator for the proximity of the Polar Front and sea-ice edge [25].

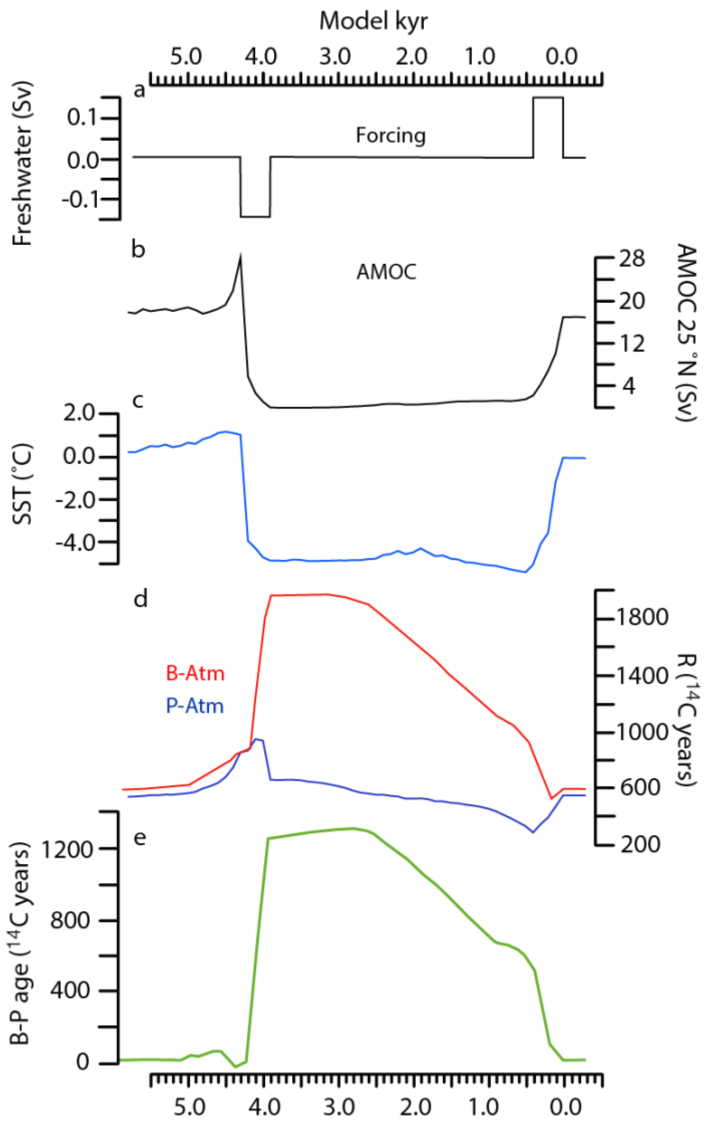

Supplementary Figure 11 – Simulated response of ^{14}C ventilation in the Nordic Seas to AMOC shutdown and recovery. a, North Atlantic freshwater forcing used to simulate AMOC reduction or shutdown and subsequent recovery. **b**, Simulated AMOC evolution at 25˚N. **c**, Sea surface temperatures in the Norwegian Sea averaged over 60-70° N and 0-10° W. **d**, Simulated bottom (1500 m) and surface (0 m) ^{14}C ventilation (R) histories in the Norwegian Sea. **e**, B-P age offset based on ventilation estimates in (d).

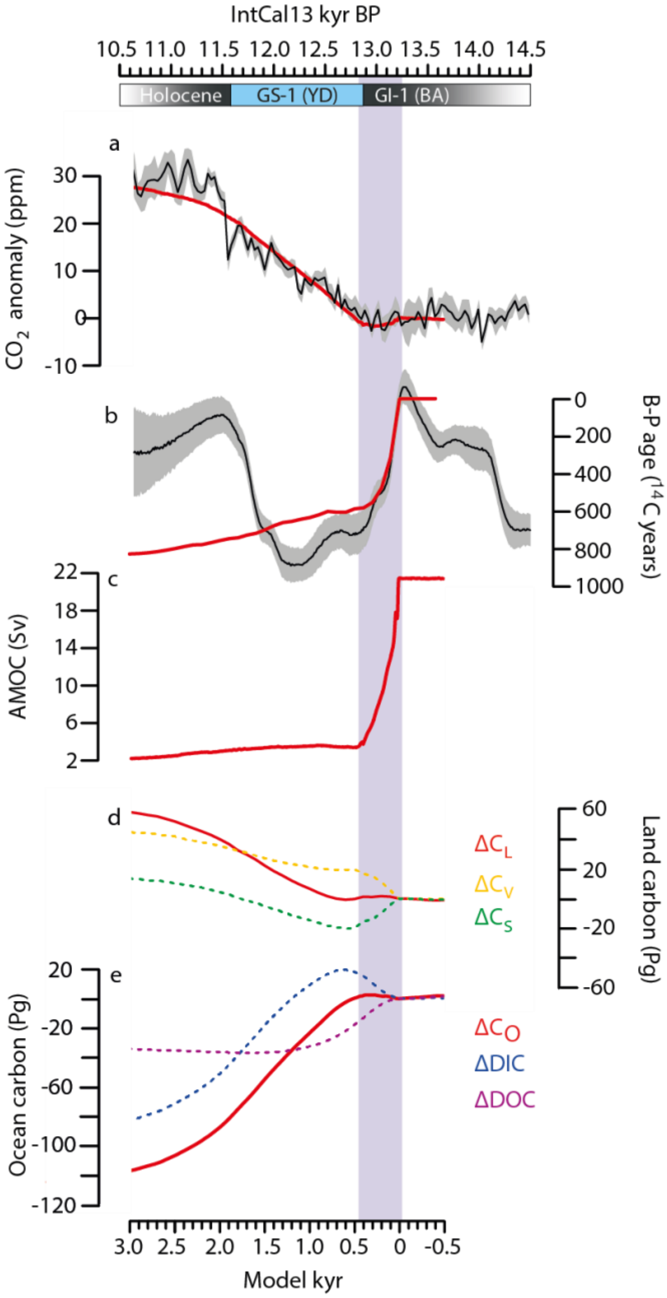

Supplementary Figure 12 – Simulated response of atmospheric CO_2 to AMOC reduction. a-b, Proxy-model comparison (proxy data in black and model results in red lines) between atmospheric CO_2 anomalies, and B-P ages at MD99-2284 coring site. Proxy data are displayed with their 68% credible intervals (1σ) associated with both analytical and chronological uncertainty in the raw data (grey envelopes) and posterior median values (black lines). Modelled B-P time series was averaged over 60-70° N and 0-10° W. Reconstructed and modelled CO_2 concentrations are presented as anomalies relative to 13000-14500 years BP and the unperturbed period prior to freshwater forcing (-500-0 model years), respectively. **c**, Simulated AMOC function in response to a stepwise 400-year-long 0.15 Sv freshwater input to the North Atlantic (45-65° N, 60-0° W). **d-e**, Simulated changes in global land and ocean carbon inventories. Changes in land carbon $\Delta C_L = \Delta C_V + \Delta C_S$ are due to vegetation ΔC_V and soil ΔC_S changes. Ocean carbon changes $\Delta C_O = \Delta DIC + \Delta DOC + \Delta POC$ are due to dissolved inorganic (DIC), organic (DOC)

and negligible changes in particulate (POC) carbon. Purple area highlights the lag between the initial AMOC weakening and the first signs of CO_2 rise recorded in the proxy data as calculated in Figure 4 (Δage: 435 ±80 years). Greenland stratigraphic events relative to the IntCal13 timescale are displayed at the top (GS: Greenland Stadial; GI: Greenland Interstadial; YD: Younger Dryas Stadial; BA: Bølling-Allerød Interstadial).

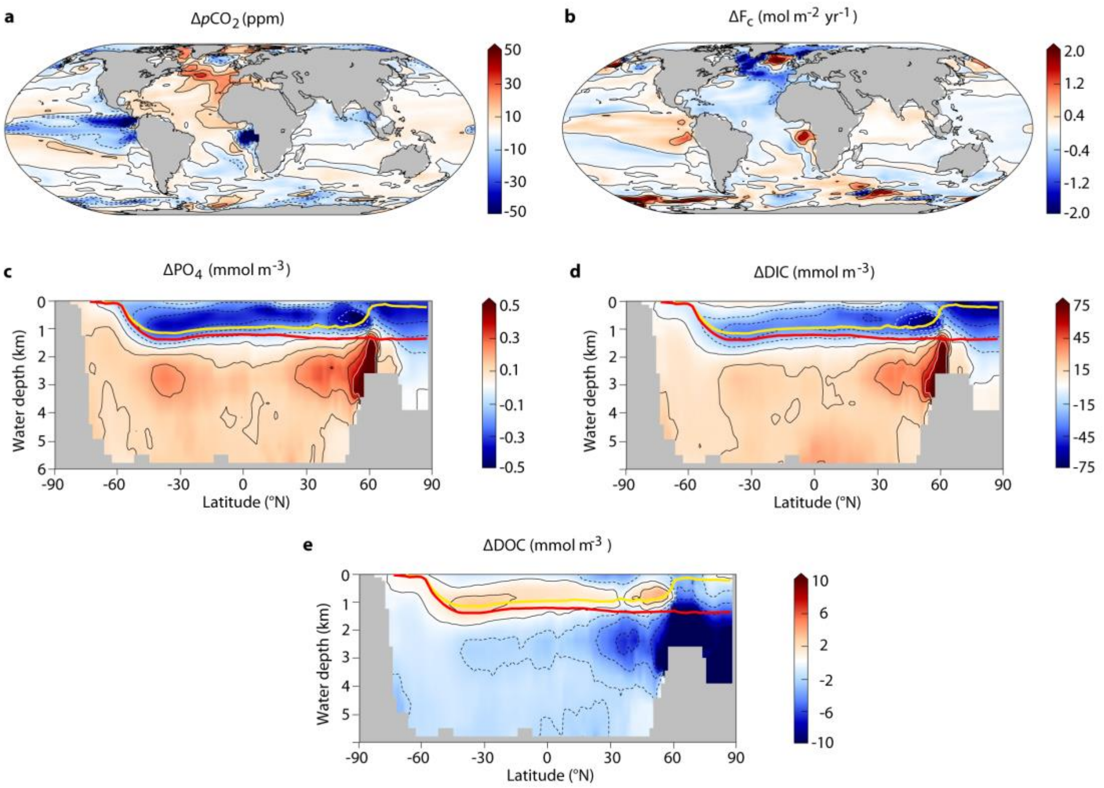

Supplementary Figure 13 – Simulated impact of AMOC weakening on oceanic pCO_2, downward carbon fluxes, nutrient availability, dissolved inorganic carbon, and dissolved organic carbon. a, Averaged surface water pCO_2 anomaly immediately after freshwater forcing relative to the unperturbed state (averages for model years 400-600 minus averages for model years -300-0). **b**, Same as in (a) for downward carbon fluxes (F_c). **c**, Zonally averaged changes in nutrients (PO_4) after freshwater forcing relative to the unperturbed state. **d**, Same as in (c) for dissolved inorganic carbon (DIC). **e**, Same as in (c) for dissolved organic carbon (DOC). The red and yellow lines show the zonally averaged potential density (σ_θ) isolines (27.5) after and prior to freshwater hosing, respectively.

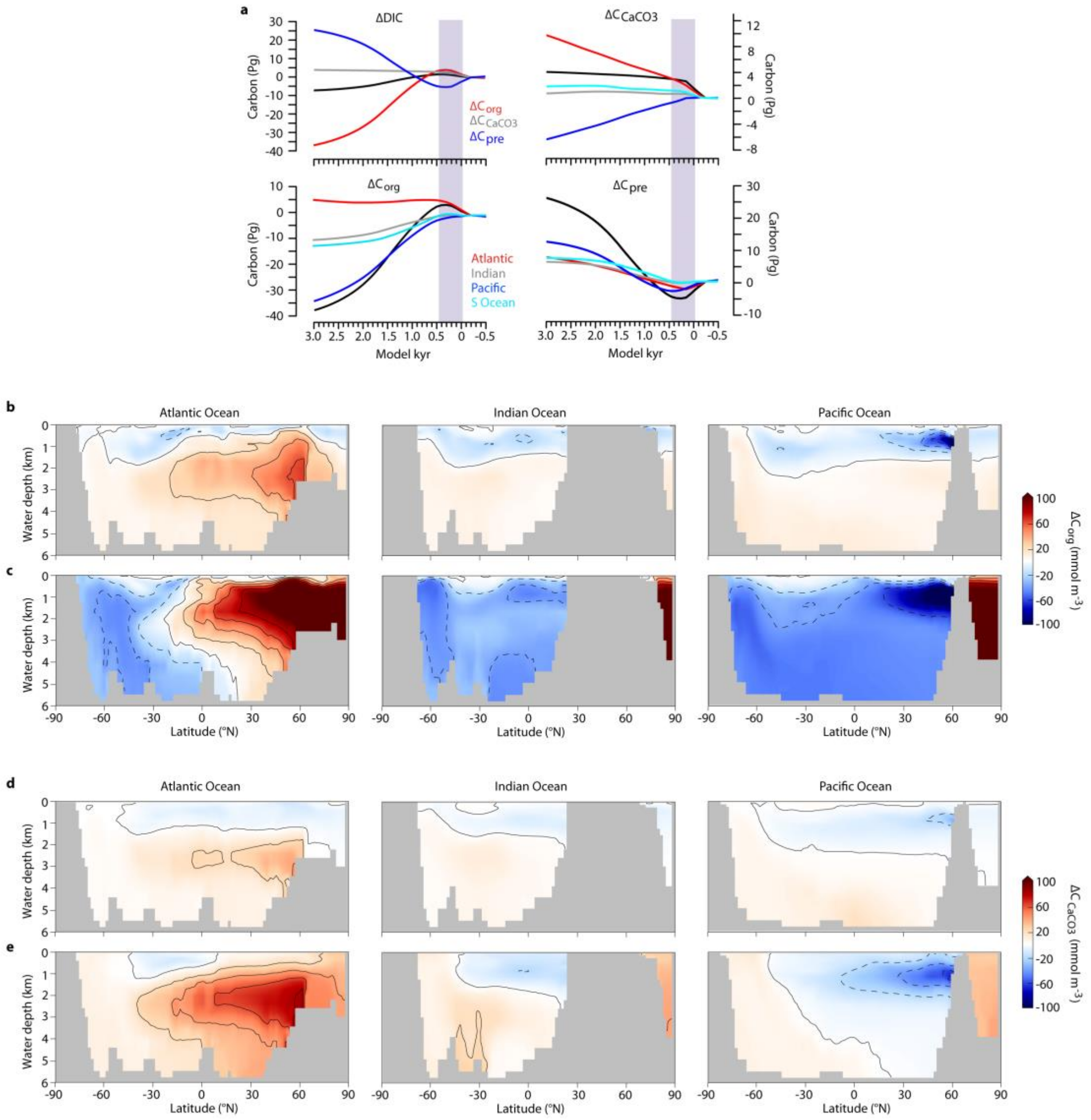

Supplementary Figure 14 – Simulated changes in ocean dissolved inorganic carbon components in response to AMOC weakening and shutdown. a, Averaged global and basin-wide changes in dissolved inorganic carbon (DIC) terms ($\Delta DIC = \Delta C_{pre} + \Delta C_{org} + \Delta C_{CaCO3}$; see ref. [26] for the calculation of the individual terms). **b**, Zonally averaged changes in organic carbon (C_{org}) in the Atlantic, Indian, and Pacific ocean basins after freshwater forcing relative to the unperturbed state (model years 400 minus model years 0). **c**, Same as in (b) but during the AMOC off state relative to the unperturbed state prior to freshwater forcing (model years 3000 minus model years 0). **d**, Same as in (b) but for calcium carbonate carbon (C_{CaCO3}). **e**, Same as in (c) but for C_{CaCO3}.

Supplementary Tables

Supplementary Table 1 – *Breakpoint ages (IntCal13 years BP) of piecewise linear ramps estimated on synchronized deglacial proxy records presented in this study (Figure 3). Estimated ages reflect start and end of each estimated ramp on the IntCal13 timescale. (I) Interstadial; (S) Stadial.*

**Proxy**	I-**S transition** (years BP **±1σ)**	**S**-I **transition** (years BP **±1σ)**
**$p CO_2$**	12814 ±134	10990 ±155
**Greenland Temperature**	12697 ±35 and 12864 ±39	11506 ±11 and 11631 ±11
**Nordic Seas NADW**	12960 ±77 and 13249 ±25	11553 ±38 and 12078 ±42
**Atmospheric $\Delta^{14}C$**	12460 ±73 and 13348 ±46	11563 ±40 and 11940 ±62

Supplementary Note 1

Suitability of the chronology

The chronology of core MD99-2284 was generated by synchronizing foraminifer fauna-based sea-surface temperatures (SST) to a hydro-climate record from Southern Sweden over the interval ~12600-15100 years BP, and by using three distinct tephra markers (see details below) over the interval ~10200-12600 years BP (Supplementary Fig. 3-4).

The hydro-climate record is based on a new leaf-wax hydrogen isotope (δD) record generated from the fossil lake of Atteköp, along the southwestern coast of Southern Sweden [6]. The site is characterised by exceptionally high sedimentation rates, which allows reconstructing δD at decadal resolution. We employed the δD composition of C_{23}-alkanes, which is a representative component of aquatic macrophyte sources [27,28]. In particular, at Atteköp C_{23}-alkanes have been assigned to a mixture of *Potamogeton* spp. and *Myriophyllum* spp., which are continuously present after ~15000 yr BP [6] and have been shown to synthesize large amounts of C_{23}-alkanes [27]. The hydrogen isotopic record of C_{23}-alkanes is here supported by an independent reconstruction of aquatic δD based on saturated C_{20} highly-branched isoprenoids (HBI), which at Atteköp are biosynthesized by epiphytic diatoms [7]. The two δD records are broadly consistent (Supplementary Fig. 3). However, due to low concentrations of HBIs in Atteköp sediments, the diatom-based δD record has a relatively lower temporal resolution than its C_{23}-alkane counterpart. For this reason, we here only use the C_{23}-alkane δD reconstruction for synchronization between our lake and marine sediment cores.

Aquatic δD records have been successfully employed in Europe for paleo-humidity reconstructions during deglaciation and are established indicators of oceanographic processes via the hydro-climate system [29,30]. Aquatic δD reflects the isotopic composition of precipitation δD, which is controlled at mid-to-high latitudes predominantly by moisture source composition [31]. As detailed in ref [6], the δD record based on C_{23}-alkanes from Atteköp can be confidently used in the period under investigation in this study as a tracer of changes in paleo-precipitation with negligible or no secondary effects from changes in terrestrial vegetation and local hydrological balance.

It has been demonstrated that hydro-climate in western Scandinavia is tightly coupled and synchronized to variability of ocean temperatures associated with the North Atlantic Current and the Norwegian Atlantic Current in the Nordic Seas [32]. More specifically, under modern conditions precipitation over the western coast of southern Sweden is delivered by the prevailing westerly winds (Supplementary Fig. 1a-b) and the amount of moisture transported to this region primarily depends on surface-water temperatures in the North and Norwegian Sea [33], which modulates water-to-air vapour fluxes (Supplementary Fig. 1a-b).

Recent studies using general circulation models [34,35] suggest that the atmospheric circulation during the last deglaciation may have been similar to today over the North Atlantic, provided that the height of the Laurentide Ice Sheet is lower than the Rocky Mountains, such as during the period analysed in this study [36]. Hence, during deglaciation moisture was likely transported from adjacent marine moisture sources under the influence of the dominant westerly winds [37,38]. This is confirmed by transient climate simulations of the last deglaciation, showing a strong correlation between surface/near-surface temperatures in the North Sea and the southern sector of the Norwegian Sea, and moisture transport to Atteköp. (Supplementary Fig. 1c-f; Supplementary Fig. 2). Therefore, we argue that our δD record is ideally suited for monitoring upwind oceanographic changes integrated as isotopic shifts in the hydrogen stable isotope composition of paleo-precipitation, and thus a reliable benchmark against which to tune appropriate regional marine proxy records spanning deglaciation.

The age-depth model for Atteköp was constructed with OxCal v4.2 (ref. [39]) using a series of 37 AMS ^{14}C dates based exclusively on terrestrial plant macrofossils and calibrated using the IntCal13 calibration curve [40]. Also included in the age model is the Hässeldalen Tephra, which was identified in Atteköp's sediments (Supplementary Fig. 3). The ^{14}C sequence was modelled in OxCal several times prescribing different k parameters until an optimal set up was obtained, i.e. until satisfactory agreement indices were obtained [39]. The final model was run using a k parameter of 0.4 suitable to constrain the ^{14}C series in a flexible way [41]. The output model yielded a coherent and solid structure of the dated sequence as defined by a good agreement index of 71.4% [42] (Supplementary Fig. 3). Unfortunately, due to the lack of terrestrial plant remains for ^{14}C dating between ~12500 and ~11500 years BP, the age model is uncertain within this interval. Nonetheless, the interval spanning ~12600-14200 years BP –which is particularly important to constrain the chronology of MD99-2284 before and during the early stage of YD– appears robust, without age reversals and with nearly linear sedimentation rates.

To assign the chronology and age of Atteköp to the proxy stratigraphy of core MD99-2284, we used a Monte Carlo algorithm for proxy-to-proxy stratigraphical alignment [7,8,24]. The method involves nonlinear deformation of the entirety of one record (SST) onto a reference record (δD) via a Monte Carlo technique that estimates an optimal alignment function. The alignment between the marine and terrestrial proxy record relies on the assumption that oceanographic changes at MD99-2284 are immediately propagated downwind and transmitted to Atteköp hydro-climatically. Indeed, at the time scales resolved by our sedimentary records (i.e. decadal and multi-decadal) we can dismiss any delay in the propagation of regional surface temperature anomalies on western Scadinavian hydro-climate [32]. However, should we consider the possibility (although unlikely) of a lagged hydro-climate response to ocean temperature changes in the Norwegian Sea, this would

not undermine our conclusions. A lagged response of δD values relative to changes in SST in fact implies that alignment between these two proxies would shift the marine records towards systematically younger ages. In turn this would mean that the leads between shifts in our B-P ages and abrupt changes in Greenland temperatures presented in this study are conservative estimates.

For the alignment process we employed a SST record from core MD99-2284 [10,43] using transfer functions based on foraminifer fauna, which we interpret here as a proxy for surface temperature conditions. However, previous investigations on the same core focussing on Marine Isotope Stage 3 (MIS3) have interpreted this record as a proxy for sub-surface or near-surface conditions [44]. During MIS3 the Nordic Seas likely had a structure similar to the modern Arctic Ocean [45], with a pronounced halocline between 50 and 200 m and thick layer of warm water sourced from the subtropical North Atlantic and centred near 300-500 m [46]. It has been suggested that under glacial conditions with a pronounced halocline in the Nordic Seas [47], *Neogloboquadrina pachyderma (s)* (NPS), which accounts for nearly 100% of the total planktic assemblage in MD99-2284 during MIS3 stadials [44], dwelled in relatively deeper and more saline waters, similarly to the modern western Nordic Seas where NPS is confined below the halocline [45]. This has led to the assumption that during MIS3, NPS in the Norwegian Sea monitored warm Atlantic Inflow waters as deep as 300-500 m, well below the mixed layer and underneath a thick halocline [44,47]. During deglaciation the background climate was warmer than MIS3 and reconstructions [47] demonstrate that the vertical structure of the Norwegian Sea was considerably different and more similar to interglacial conditions. We therefore argue that in the absence of a thick halocline, NPS –which during deglaciation almost entirely dominates the total planktic assemblage that drives the temperature signal in our record (Supplementary Figs. 3, 10)– occupied the mixed layer at shallower depths very close to the sea surface (20-50 m), like in the Arctic domain of the modern Nordic Seas [45]. This seems likely especially for the relatively warmer GI-1 interval and early GS-1 given that recent studies from Fram Strait [48] have shown that today NPS can thrive above 100 m even in the presence of sea ice or in proximity of the ice edge. Moreover, our interpretation is justified by the extensive use of NPS in this region for constructing Greenland tuned age models spanning deglaciation. In fact, a classical approach involves defining time boundaries based on the assumption that abrupt an warming/cooling in Greenland, as recorded in the $\delta^{18}O$ stratigraphies, corresponds to a synchronous sea surface/subsurface warming/cooling based on NPS $\delta^{18}O$ or %NPS (e.g. ref. [15,49,50]). Finally, we should note that our proxy-to-proxy alignment strategy and the resulting chronology is independent on whether we use SST or %NPS data, as the related records are virtually the same (Supplementary Fig. 3).

To test the sensitivity of our B-P ages to the underlying chronology, we developed an alternative age-depth model that leans on the use of stratigraphic tie points

based on correlation between %NPS and NGRIP $\delta^{18}O$, as described above. The Greenland tuned age model includes 5 tie points, in addition to the 3 tephra horizons previously identified in core MD99-2284 (and discussed in the section below). The results from the Greenland tuned age model are shown in Supplementary Figure 4. Using an ice-core based chronology does not affect our main conclusions, i.e. that B-P ages increase significantly earlier than the onset of GS-1 and cancel out significantly earlier than the onset of the Holocene. As we can measure B-P ages and surface temperature conditions in the same sedimentary record, we can state that B-Atm ages become significantly older than P-Atm ages shortly prior to a warming event (that is an abrupt drop in %NPS) just preceding the cold GS-1 stadial. Analogously, B-Atm age equals P-Atm values during a cooling event (that is an abrupt increase in %NPS) just prior to the start of the warm Holocene interglacial. This observation is independent of the approach used to construct the chronology and show unequivocally that aging (youngening) of intermediate/deep water in the Norwegian sea lead ahead surface water cooling (warming).

On a final note, we observe that sedimentation rates in MD99-2284, as inferred from the chronology presented in this study, increase during the second half of GS-1. This change in accumulation rates is not surprising. The second half of GS-1 was characterised by a northern shift of the North Atlantic storm tracks, which have been recorded both in Greenland ice cores and European lake sediment reconstructions [10,51]. Glacier mass turnover records from the well-dated site of Lake Kråkenes, on the western coast of Norway [18], show that the northward drift of the westerly winds destabilised regional glaciers by enhancing both winter ice-sheet growth and summer melting (Supplementary Fig. 3). This destabilization, which is also evidenced by a faster rate of sedimentation rate above the Vedde Ash at Kråkenes [10], likely led to higher calving and runoff rates off the Fennoscandian Ice Sheet. Increased input of terrigenous debris, meltwater and IRD –in conjunction with faster sedimentation rates (~5 times higher) is evident above the Vedde Ash in core MD99-2284 (Supplementary Figs. 3, 7). Other high-resolution cores from the northern sector of the Norwegian Sea [20] (Supplementary Fig. 7) are consistent with our records, documenting a major rise in IRD fluxes after the Vedde Ash, accompanied by an increase in sedimentation rates (~4.5 times higher). Enhanced melting during GS-1 has also been observed in relation to the Greenland and Laurentide Ice Sheet [52], thus confirming that iceberg and meltwater discharge of northern hemisphere ice sheets were decoupled from the dominant North Atlantic climate mode.

Supplementary Note 2

Tephra measurements and implications

In core MD99-2284 the Vedde Ash and the Abernethy Tephra occur as two well defined and distinct peaks about 115 cm apart (Supplementary Fig. 4). Given the extremely high sedimentation rates (> 400 cm kyr^{-1}) at our study site, we can rule out that the younger tephra is a result of bioturbation in the mixed sediment layer. Reworking of the earlier deposited Vedde Ash is also unlikely for a number of reasons: *i*) the two tephra horizons are stratigraphically discrete, single layer, and structured as two very sharp and well defined peaks in shard concentration, similar to other distal ash layers that have been considered primary deposits; *ii*) concentration of shards in the range 150-500 µm (fine to medium sand) are less than 1% and do not correlate with tephra concentrations; *iii*) the disappearance of coarse-grained clasts associated with ice-rafted debris (>0.5 mm) about 30 cm below the upper tephra rules out secondary transport mechanisms via ice rafting; *iv*) the upper ash layer is not accompanied by any peak in terrestrial long-chain *n*-alkanes that may suggest reworking at the sediment source input (Figure 2). In addition, the upper tephra layer is detected at the base of the overlying Early Holocene warming documented by a major drop in the relative abundance of *Neogloboquadrina pachyderma (s)* in MD99-2284 (Supplementary Fig. 3). This is stratigraphically consistent with other terrestrial records whereby the Abernethy Tephra has been identified very closely to the YD/Holocene boundary [53]. Altogether, these lines of evidence support the proposal that the two ash layers represent two separate eruptive events. In fact, it should be born in mind that even though the two tephra layers have virtually identical geochemical signatures, it is well established that the Katla system generated widespread ash layers with comparable glass shard composition during deglaciation [12]. For instance, the Dimma Ash and Suduroy Tephra, which occurred during the Oldest Dryas and Early Holocene respectively, both hailed from the Katla volcanic system and are compositionally coherent with Vedde Ash deposits [12]. The Katla is not a unique volcanic system in this regard and ash deposits with identical major element chemical signatures are also generated from other Icelandic volcanoes [54].

Supplementary Note 3

Simulated impact of AMOC slowdown on pCO$_2$ rise

Our B-P age reconstructions suggest that atmospheric pCO$_2$ rise lags behind weakening of an important component of NADW at the onset of GS-1 by 437 ±79 years (Fig. 3). Although a lag between AMOC weakening and pCO$_2$ rise has been previously hypothesised [55,56], our study is the first to our knowledge that provides a precise estimation of this time lag that does not depend on Greenland tuning or marine ^{14}C chronologies relying on assumptions about local reservoir ages. To explore the mechanisms behind the lagged response time of the carbon cycle to a weakening of NADW production in the North Atlantic and AMOC, we analyse

results from climate model experiment with an Earth System Model including climate-biogeochemistry interactions described in detail in ref. [26].

In the simulations, which are initialized from a preindustrial equilibrium, the AMOC was reduced or shut down due to freshwater forcing in the North Atlantic (Supplementary Fig. 11-12). In particular, in this study we only refer to the simulations that were conducted using freshwater amplitude of 0.15 Sv. It should be noted that our model experiments are highly idealised and only aimed at understanding the drivers of a delayed pCO_2 rise in response to NADW weakening inferred from the proxy reconstructions presented in this study. The experiments were performed with the University of Victoria Earth System Climate Model (UVic ESCM) version 2.9, which consists of a three-dimensional ocean general circulation model at coarse resolution (1.8x3.6 deg, 19 vertical levels), a dynamic-thermodynamic sea ice model, a single-layer Energy-Moisture-Balance-Model of the atmosphere as well as land surface and ocean biogeochemistry. The Model of Ocean Biogeochemistry and Isotopes (MOBI) version 1.4 includes nitrate and phosphate as inorganic nutrients, two phytoplankton functional groups (diazotrophs and other phytoplankton), zooplankton, detritus, dissolved organic matter [57], dissolved inorganic carbon (DIC), and alkalinity. ^{13}C isotopes are tracked in all carbon components considering fractionation during photosynthesis and air-sea gas exchange. ^{14}C is implemented by duplicating DIC and adding radioactive decay. Thus, fractionation during air-sea exchange and photosynthesis are not considered for ^{14}C because their effects are much smaller than those of radioactive decay. The model has been shown to successfully reproduce large-scale biogeochemical tracer distributions including radiocarbon in the modern ocean [58,59].

Both the simulated rate of pCO_2 rise and the change in deep-water ventilation in the Norwegian Sea agree well with the long-term trend measured in Antarctic ice cores and B-P ages observed in MD99-2284, respectively (Supplementary Fig. 11-12). The simulations show that the pCO_2 rise lags the first signs of AMOC weakening by ~400 years, a result that is consistent with our observed 437-year lag (Fig. 4), and that is also modelled under glacial boundary conditions [60]. The simulated response time between AMOC weakening and pCO_2 rise is similar in experiments conducted using a larger freshwater amplitude [26]. A recent study using a different coupled climate-carbon models of intermediate complexity [61], consistently simulated a ~400 year lag across a suite of similarly designed sensitivity experiments using freshwater amplitudes higher than 0.1 Sv. The same study showed that the absolute timing of atmospheric pCO_2 rise depends on the duration of the prescribed freshwater forcing. However, all the sensitivity experiments using different durations of a 0.2 Sv freshwater pulse, indicate a consistent lag of a few centuries between NADW export minimum and the first signs of pCO_2 rise. This suggest that the simulated lag is independent of the

duration and amplitude of the hosing, provided that the freshwater is higher than the model-characteristic threshold value beyond which NADW cannot be sustained [62].

The simulated lag observed in our model is largely driven by reduced upwelling of nutrient-rich deep waters in the Southern Ocean in response to AMOC slowdown [63] (Supplementary Fig. 12-13). Reduced upwelling causes a decline in upper ocean nutrient levels, which decreases global phytoplankton stocks and dissolved organic carbon (DOC) production and concentrations [60,63]. Decreasing DOC compensates for rising ocean DIC until about year 400, which is dominated by the accumulation of biologically sequestered carbon (C_{org} and C_{CaCO3}) in the subsurface and deep North Atlantic (Supplementary Fig. 14). After year 400, DIC levels off and eventually declines due to a gradual loss of C_{org} in the deep Pacific, Indian, and Southern Oceans (Supplementary Fig. 14) in response to a reduction in the global efficiency of the biological pump, ultimately leading to a decrease in the total ocean carbon content. The momentary balance between decreasing DOC and increasing DIC results in a lag in the global response of atmospheric $p\mathrm{CO}_2$ rise to slowing AMOC (Supplementary Fig. 12-13).

Supplementary Methods

Estimating Reservoir Ages

Consider a set of N ocean objects with calendar ages $\theta_1, \ldots, \theta_N$and unknown *site-specific* ocean radiocarbon ages $c_{ocean}(\theta_1), \ldots, c_{ocean}(\theta_N)$. We do not know these values exactly but observe them subject to noise i.e. we observe pairs $(t_i, z_i)_{i=1,\ldots,N}$ with

$$t_i = \theta_i + \epsilon_i$$
$$z_i = c_{ocean}(\theta_i) + \eta_i,$$

where ϵ_i and η_i are the uncertainties in our measurement of calendar age and radiocarbon age. In this paper we assume these to be independent and identically distributed (IID) with mean 0 and variances τ_i^2 and σ_i^2 respectively although more general (and non-independent) uncertainties are also permissible (e.g. ages estimated by varve counting). Decomposing the *site-specific* ocean radiocarbon age as

$$c_{ocean}(\theta_i) = m(\theta_i) + \Delta R(\theta_i),$$

where $m(\theta_i)$ is the *site-general* marine calibration age and $\Delta R(\theta_i)$ the local reservoir variation i.e. difference between the reservoir age at the specific site of interest and that implicitly used for the site general $m(\cdot)$, we are left with the following model,

$$t_i = \theta_i + \epsilon_i$$
$$z_i = m(\theta_i) + \Delta R(\theta_i) + \eta_i.$$

Our aim is to estimate $\Delta R(\theta)$ from our paired (t_i, z_i) observations and our prior knowledge of $m(\theta)$ provided by the marine calibration curve; and hence infer $R(\theta)$ the absolute marine reservoir in the Norwegian Sea. This is done via a Metropolis-within-Gibbs Bayesian approach as described below.

Priors on the unknown variables

Prior on θ_i

We place an improper flat prior on each θ_i i.e. $\pi(\theta_i) = 1$. This diffuse prior represents our lack of prior knowledge about the true calendar age of the object until we perform a radiocarbon determination upon it.

Prior on $m(\theta)$

In order for the above problem to be identifiable, we need to have considerable prior information on the value of the *site-general* $m(\theta)$. This is provided by the Marine13 radiocarbon calibration curve [40]. This provides pointwise estimates of its mean $\mu(\theta)$ and variance $\sigma_c^2(\theta)$ on a grid. We place a prior

$$m(\theta) \sim N\big(\mu(\theta), \sigma_c^2(\theta)\big).$$

Values for θ not lying on the marine calibration grid can be found using interpolation.

Prior on $\Delta R(\theta)$

As described in the main paper, we place a random walk prior to model the evolution of the site-specific $\Delta R(\theta_i)$

$$\Delta R(\theta) \mid \Delta R(\theta^*) \ \sim N(\Delta R(\theta^*), \rho|\theta - \theta^*|),$$

i.e. given the local variation $\Delta R(\theta^*)$ (at a time θ^*) then we might think that the value $\Delta R(\theta)$ at a neighbouring time θ would likely also be around $\Delta R(\theta^*)$. This random walk prior model is analogous to that used in the modelling of the atmospheric calibration curve IntCal13.

Here the parameter ρ^2 determines how much we expect the local reservoir to vary from one year to the next. This is a crucial parameter in determining the wiggliness of the final curve. We therefore place a hierarchical prior on its value

$$\frac{1}{\rho^2} \sim Gamma(\alpha, \beta)$$

and update it within our sampler. Discussion on the suitable selection of the hyper-parameters α, β is provided later.

Metropolis-within-Gibbs Sampler

We update our unknown variables using a Metropolis-within-Gibbs sampler as in ref. [64–66] although here we are required to also include the unknown $m(\theta)$. The approach taken is to update in turn:

1. The pairs $(\theta_i, \Delta R(\theta_i))$ for $i = 1, \dots, N$;
2. The *site-general* marine calibration ages $m(\theta_i)$ for $i = 1, \dots, N$;
3. The variance ρ^2 of our random walk modelling the local reservoir age variation.

Below the full vector for a variable is shown in bold type e.g. $\boldsymbol{X} = (x_1, \dots, x_n)^T$ while e.g. $\boldsymbol{X^{-i}} = (x_1, \dots, x_{i-1}, x_{i+1}, \dots, x_n)^T$ denotes the full vector excluding the ith term.

<u>Updating $(\theta_i, \Delta R(\theta_i)) \mid \boldsymbol{T}, \boldsymbol{Z}, \boldsymbol{\theta^{-i}}, \boldsymbol{\Delta R^{-i}}, \boldsymbol{m}, \rho^2$</u>

We use Metropolis-Hastings to update each pair $(\theta_i, \Delta R(\theta_i))$. Creating dummy variables,

$$x_i = z_i - m(\theta_i),$$

this step becomes identical to that detailed in ref. [64,65]. We refer to these papers for details.

<u>Updating $m(\theta_i) \mid \boldsymbol{T}, \boldsymbol{Z}, \boldsymbol{\theta}, \boldsymbol{\Delta R}, \boldsymbol{m^{-i}}, \rho^2$</u>

First note

$$p(m(\theta_i) \mid \boldsymbol{T}, \boldsymbol{Z}, \boldsymbol{\theta}, \boldsymbol{\Delta R}, \boldsymbol{m^{-i}}, \rho^2) = p(m(\theta_i) \mid z_i, \theta_i, \Delta R(\theta_i))$$
$$\propto p\big(z_i \big| m(\theta_i), \Delta R(\theta_i)\big) p(m(\theta_i) \mid \theta_i, \Delta R(\theta_i)).$$

Then since,

$$z_i - \Delta R(\theta_i) \mid m(\theta_i) \sim N(m(\theta_i, \sigma_i^2),$$
$$m(\theta_i) \sim N\big(\mu(\theta_i), \sigma_c^2(\theta_i)\big),$$

we have a conjugate posterior

$$m(\theta_i) \mid \boldsymbol{T}, \boldsymbol{Z}, \boldsymbol{\theta}, \boldsymbol{\Delta R}, \boldsymbol{m^{-i}}, \rho^2 \sim N\big(\mu'(\theta_i), \sigma'^2(\theta_i)\big),$$

where

$$\sigma'^2(\theta_i) = \frac{1}{\sigma_i^{-2} + \sigma_c^{-2}(\theta_i),}$$
$$\mu'(\theta_i) = \sigma'^2(\theta_i)\left(\frac{\mu(\theta_i)}{\sigma_c^2(\theta_i)} + \frac{z_i - \Delta R(\theta_i)}{\sigma_i^2}\right).$$

<u>Updating $\rho^2 \mid \boldsymbol{T}, \boldsymbol{Z}, \boldsymbol{\theta}, \boldsymbol{\Delta R}, \boldsymbol{m}$</u>

It is sufficient to consider the normalised increments

$$\tilde{d}_i = \frac{\Delta R(\theta_{i+1}) - \Delta R(\theta_i)}{\sqrt{\theta_{i+1} - \theta\_i}} \qquad for\ i = 1, \dots, N-1$$

Since $\tilde{d}_i \mid \rho^2$ are IID $N(0, \rho^2)$, with our choice of prior

$$\frac{1}{\rho^2} \mid \boldsymbol{T}, \boldsymbol{Z}, \boldsymbol{\theta}, \boldsymbol{\Delta R}, \boldsymbol{m} \sim Gamma(\alpha', \beta'),$$

where

$$\alpha' = \alpha + \frac{N-1}{2},$$
$$\beta' = \beta + \frac{\sum_{i=1}^{N-1} \tilde{d}_i^2}{2}.$$

Choice of $\boldsymbol{\alpha}, \boldsymbol{\beta}$ in prior on $\boldsymbol{\Delta R}$

The values of α, β chosen within our random walk model for the reservoir age encode how variable we believe this function to be over time through the prior on ρ^2. To select suitable values we incorporated our prior beliefs on how much one might expect the site reservoir age to change over a 10 year period.

Under our Weiner process prior model, the difference in the value of the reservoir age over any 10 year period $\Delta R(\theta + 10) - \Delta R(\theta) \sim N(0, 10\rho^2)$ and so, given ρ^2, a 95% predictive interval for the difference in the reservoir age over the course of a 10 year period would be $\pm 2\sqrt{10\rho^2}$. Given α, β, the expected value of the random walk variance $E[\rho^2] = {}^{\beta}/_{\alpha - 1}$. Substituting this in (i.e. approximating $E[\rho]$ by $\sqrt{E[\rho^2]}$) implies that a specific α, β approximately equates to a 95% belief that the change in reservoir age over a 10 year period would be less than $\pm 2\sqrt{10\frac{\beta}{\alpha-1}}$.

Consideration of plausible reservoir age changes therefore allows selection of α, β to match with those beliefs. Furthermore, the smaller α the more uncertain you are about the value of the random walk variance, the larger the more certain. All the curves presented in this study were generated using values of α= 5 and β= 80, which correspond to a prior estimate for the variability of the reservoir age over 10 years of ~30 years.

Interpolating output from current calendar age estimates $\boldsymbol{\theta}$ onto a general calendar age grid

On each iteration of our MCMC sampler, we update several variables of potential interest:

- $\boldsymbol{\theta}$ – the estimated calendar ages of the ocean objects;
- $\boldsymbol{\Delta R(\theta)}$ – the difference between the site-specific and site-general reservoir ages;
- $\mathbf{m(\theta)}$ – an updated estimate of the site-general marine calibration age;
- ρ – the smoothing parameter on the $\Delta R(\theta)$ random walk.

The nature of our sampler means that the second and third variables (i.e. $\Delta R(\theta)$ and $m(\theta)$ on this list are only provided at the current values of $\boldsymbol{\theta}$ for that MCMC iteration (i.e. the current estimates for the calendar ages of ocean objects). Generally however, we would wish to estimate these two functions at any calendar

age on a grid of our choosing. This requires some additional processing described below.

In the case of $\Delta R(\theta)$ this step is relatively straightforward due to the properties of our Weiner process random walk. Suppose that the current iteration of the MCMC sampler has provided estimates of the calendar ages of our ocean objects $\boldsymbol{\theta}$; estimates $\boldsymbol{\Delta R}(\boldsymbol{\theta})$ of the site-specific reservoir difference at these ages; and a value for the random walk smoothness ρ. Now consider that we want to estimate the reservoir age difference ΔR at θ^*, a calendar age of our choosing. Due to the random walk prior on $\Delta R(\theta)$ we find that estimating the reservoir age at any potentially new point of interest θ^* simplifies to solving a Brownian bridge conditional upon the value of ΔR at those values of $\boldsymbol{\theta}$ directly above and below θ^*:

$$\begin{aligned}[\,\Delta R(\theta^*)|\ \boldsymbol{T}, \boldsymbol{Z}, \boldsymbol{\theta}, \boldsymbol{\Delta R}, \boldsymbol{m}, \boldsymbol{\rho^2}] &= [\,\Delta R(\theta^*)|\ \boldsymbol{\theta}, \boldsymbol{\Delta R}, \rho^2] \\ &\propto [\,\Delta R(\theta^*)|\ \Delta R(\theta^A), \Delta R(\theta^B), \theta^A, \theta^B, \rho^2]\end{aligned}$$

Here θ^A and θ^B are the individual elements of $\boldsymbol{\theta}$ that lie directly above and below θ^* respectively and bound the Brownian bridge. Hence, excluding values of θ^* that lie above/below all elements in $\boldsymbol{\theta}$ for which we only have a single bound to our bridge, we find

$$\begin{aligned}&\Delta R(\theta^*)|\ \Delta R(\theta^A), \Delta R(\theta^B), \theta^A, \theta^B, \rho^2 \\ &\qquad\sim N\left(\Delta R(\theta^B) + \phi\big(\Delta R(\theta^A) - \Delta R(\theta^B)\big), \rho^2(\theta^A - \theta^B)\phi(1-\phi)\right),\end{aligned}$$

where $\phi = \frac{\theta^* - \theta^B}{\theta^A - \theta^B}$. To build up realisations of $\Delta R(\theta)$ on a pre-determined grid of calendar ages given the MCMC output we can therefore begin with the smallest calendar age in the grid and build up the realisation sequentially at increasing ages by sampling from successive Brownian bridges bounded below/above by the already realised value of ΔR at the two calendar ages lying below/above the age to be added (these bounds can come from gridded θ values already added to the realisation or values $\boldsymbol{\theta}$ from the MCMC sampler).

In the case of $m(\theta)$, we would hope to perform a similar approach to transfer from the irregular and iteration dependent MCMC calendar ages $\boldsymbol{\theta}$ onto a regular grid since, underlying the marine calibration curve, is a belief that this function too has a random walk prior (with a drift of 1). However two issues complicate the matter. Firstly, we have significant additional information about the value of the marine calibration curve at any value θ^* beyond just the outputted MCMC estimates of m at the calendar ages of the objects $\boldsymbol{\theta}$. This prior information comes from the marine calibration curve. We cannot therefore just interpolate the walk at a new point θ^* by forming a Brownian bridge bounded by these values as doing so would ignore this strong prior information. Secondly we do not have an estimate of the smoothness of the $m(\theta)$ random walk. These two factors mean that we must instead make an approximation for interpolation. We modify the approach described above by first placing a prior on the site-general $m(\theta^*)$ taken from the

published marine calibration curve. Then, to interpolate from the given MCMC output $\boldsymbol{m}$ and $\boldsymbol{\theta}$, we form a Brownian bridge as described above and combine this with the prior to give a posterior estimate for $m(\theta^*)$. The random walk variance used in the bridge for each iteration of the sampler is estimated by maximum likelihood from the MCMC output $\boldsymbol{m}$ and $\boldsymbol{\theta}$ in that iteration.

Other functions of interest

By performing the above regridding of $m(\theta)$ we are able not only to estimate $\Delta R(\theta)$ but also other functions of interest, for example the radiocarbon age of the specific site ($c_{ocean}(\theta) = m(\theta) + \Delta R(\theta)$; or the absolute reservoir age of the site – the difference between $c_{ocean}(\theta)$ and the atmospheric ^{14}C (i.e. IntCal13) from the same time.

Other modelling approaches

Other decompositions and modelling approaches are possible. For example, we could estimate $c_{ocean}(\theta)$ by refitting a random walk directly to the raw z_i values, ignoring its decomposition into $\Delta R(\theta)$ and $m(\theta)$. However, such an approach would not model the roughness of the curves equivalently – the approach described in this paper models the roughness of $\Delta R(\theta)$ i.e. penalising the local reservoir variation from the marine calibration curve; conversely fitting a random walk to the raw z_is models the roughness of $c_{ocean}(\theta)$. The estimate of $c_{ocean}(\theta)$ found by refitted a separate random walk to the z_is would therefore not be consistent with our estimates for $\Delta R(\theta)$ found above. Since we felt that decomposing $c_{ocean}(\theta)$ into $\Delta R(\theta)$and $m(\theta)$ had a stronger physical justification, with the local variation $\Delta R(\theta)$ plausibly modelled as an independent random walk.

We could also have decomposed our observations into the atmospheric values and the absolute reservoir age of the Norwegian Sea i.e.

$$z_i = n(\theta_i) + R(\theta_i) + \eta_i,$$

where $n(\theta)$ is the atmospheric radiocarbon which we have prior information on via IntCal13. This would allow us to estimate $R(\theta)$ directly but we would have to post-process analogously to above to obtain an estimate for $\Delta R(\theta)$. Investigation showed that this provides slightly differing estimates due to the box-model employed to create MarineCal that smears out small scale variation however our conclusions remain the same.

References

1. Willett, K. M., Gillett, N. P., Jones, P. D. & Thorne, P. W. Attribution of observed surface humidity changes to human influence. *Nature* **449,** 710–712 (2007).

2. Levitus, S. *et al.* The World Ocean Database. *Data Sci. J.* **12,** WDS229-WDS234 (2013).

3. Kalnay, E. *et al.* The NCEP/NCAR 40-year reanalysis project. *Bull. Am. Meteorol. Soc.* **77,** 437–471 (1996).

4. Menviel, L., Timmermann, A., Timm, O. E. & Mouchet, A. Deconstructing the Last Glacial termination: The role of millennial and orbital-scale forcings. *Quat. Sci. Rev.* **30,** 1155–1172 (2011).

5. Liu, Z. *et al.* Transient Simulation of Last Deglaciation with a New Mechanism for Bolling-Allerod Warming. *Science.* **325,** 310–314 (2009).

6. Wohlfarth, B. *et al.* Climate and environment in southwest Sweden 15.3 – 11.3 cal. ka BP. *Boreas* (2018).

7. Muschitiello, F., Andersson, A., Wohlfarth, B. & Smittenberg, R. H. The C20 highly branched isoprenoid biomarker - A new diatom-sourced proxy for summer trophic conditions? *Org. Geochem.* **81,** 27–33 (2015).

8. Muschitiello, F. *et al.* Fennoscandian freshwater control on Greenland hydroclimate shifts at the onset of the Younger Dryas. *Nat. Commun.* **6,** 8939 (2015).

9. Rasmussen, C. E. & Williams, C. K. I. *Gaussian processes for machine learning. International journal of neural systems* **14,** (2004).

10. Bakke, J. *et al.* Rapid oceanic and atmospheric changes during the Younger Dryas cold period. *Nat. Geosci.* **2,** 202–205 (2009).

11. Lowe, J. J. *et al.* The RESET project: Constructing a European tephra lattice for refined synchronisation of environmental and archaeological events during the last c. 100 ka. *Quat. Sci. Rev.* **118,** 1–17 (2015).

12. Lane, C. S. *et al.* Was the 12.1ka Icelandic Vedde Ash one of a kind? *Quat. Sci. Rev.* **33,** 87–99 (2012).

13. Matthews, I. P. *et al.* New age estimates and climatostratigraphic correlations for the borrobol and penifiler tephras: Evidence from Abernethy Forest, Scotland. *J. Quat. Sci.* **26,** 247–252 (2011).

14. Erdman, C. & Emerson, J. W. bcp : An R Package for Performing a Bayesian Analysis of Change Point Problems. *J. Stat. Softw.* **23,** 1–13 (2007).

15. Ezat, M. M. *et al.* Ventilation history of Nordic Seas overflows during the

last (de)glacial period revealed by species-specific benthic foraminiferal 14C dates. *Paleoceanography* **32,** 172–181 (2017).

16. Adolphi, F. *et al.* Persistent link between solar activity and Greenland climate during the Last Glacial Maximum. *Nat. Geosci.* **7,** 662–666 (2014).

17. Rasmussen, S. O. *et al.* A new Greenland ice core chronology for the last glacial termination. *J. Geophys. Res. Atmos.* **111,** (2006).

18. Sigl, M. *et al.* The WAIS Divide deep ice core WD2014 chronology - Part 2: Annual-layer counting (0-31 ka BP). *Clim. Past* **12,** 769–786 (2016).

19. Muscheler, R., Adolphi, F. & Knudsen, M. F. Assessing the differences between the IntCal and Greenland ice-core time scales for the last 14,000 years via the common cosmogenic radionuclide variations. *Quat. Sci. Rev.* **106,** 81–87 (2014).

20. Ebbesen, H. & Hald, M. Unstable Younger Dryas climate in the northeast North Atlantic. *Geology* **32,** 673–676 (2004).

21. Cabedo-Sanz, P., Belt, S. T., Knies, J. & Husum, K. Identification of contrasting seasonal sea ice conditions during the Younger Dryas. *Quat. Sci. Rev.* **79,** 74–86 (2013).

22. Meland, M. Y., Dokken, T. M., Jansen, E. & Hevrøy, K. Water mass properties and exchange between the Nordic seas and the northern North Atlantic during the period 23-6 ka: Benthic oxygen isotopic evidence. *Paleoceanography* **23,** (2008).

23. Dreger, D. Decadal to Centennial Scale Sediment Records of Ice Advance on the Barents Shelf and Meltwater Discharge Into the Northeastern Norwegian Sea Over the Last 40 Kyr Dekadische Bis Jahrhundert-Variabilität Von Eisvorstößen Auf Dem Barentsschelf und Schmelzwas. (1999).

24. Muschitiello, F. Deglacial impact of the Scandinavian Ice Sheet on the North Atlantic climate system. (Stockholm University, 2016).

25. Polyak, L. & Mikhailov, V. Post-glacial environments of the southeastern Barents Sea: foraminiferal evidence. *Geol. Soc. London, Spec. Publ.* **111,** 323–337 (1996).

26. Schmittner, A. & Lund, D. C. Early deglacial Atlantic overturning decline and its role in atmospheric CO2 rise inferred from carbon isotopes (δ13C). *Clim. Past* **11,** 135–152 (2015).

27. Aichner, B., Herzschuh, U., Wilkes, H., Vieth, A. & Böhner, J. δD values of n-alkanes in Tibetan lake sediments and aquatic macrophytes - A surface sediment study and application to a 16ka record from Lake Koucha. *Org. Geochem.* **41,** 779–790 (2010).

28. Gao, L., Hou, J., Toney, J., MacDonald, D. & Huang, Y. Mathematical modeling of the aquatic macrophyte inputs of mid-chain n-alkyl lipids to lake sediments: Implications for interpreting compound specific hydrogen isotopic records. *Geochim. Cosmochim. Acta* **75,** 3781–3791 (2011).

29. Rach, O., Brauer, A., Wilkes, H. & Sachse, D. Delayed hydrological response to Greenland cooling at the onset of the Younger Dryas in western Europe. *Nat. Geosci.* **7,** 109–112 (2014).

30. Rach, O., Kahmen, A., Brauer, A. & Sachse, D. A dual-biomarker approach for quantification of changes in relative humidity from sedimentary lipid D/H ratios. *Clim. Past* **13,** 741–757 (2017).

31. Gat, J. R. Oxygen and Hydrogen Isotopes in the Hydrologic Cycle. *Annu. Rev. Earth Planet. Sci.* **24,** 225–262 (1996).

32. Årthun, M. *et al.* Skillful prediction of northern climate provided by the ocean. *Nat. Commun.* **8,** (2017).

33. Gustafsson, M., Rayner, D. & Chen, D. Extreme rainfall events in southern Sweden: Where does the moisture come from? *Tellus, Ser. A Dyn. Meteorol. Oceanogr.* **62,** 605–616 (2010).

34. Löfverström, M., Caballero, R., Nilsson, J. & Kleman, J. Evolution of the large-scale atmospheric circulation in response to changing ice sheets over the last glacial cycle. *Clim. Past* **10,** 1453–1471 (2014).

35. Löfverström, M., Caballero, R., Nilsson, J. & Messori, G. Stationary Wave Reflection as a Mechanism for Zonalizing the Atlantic Winter Jet at the LGM. *J. Atmos. Sci.* **73,** 3329–3342 (2016).

36. Gowan, E. J., Tregoning, P., Purcell, A., Montillet, J. P. & McClusky, S. A model of the western Laurentide Ice Sheet, using observations of glacial isostatic adjustment. *Quat. Sci. Rev.* **139,** 1–16 (2016).

37. Isarin, R. F. B., Renssen, H. & Koster, E. A. Surface wind climate during the Younger Dryas in Europe as inferred from aeolian records and model simulations. *Palaeogeogr. Palaeoclimatol. Palaeoecol.* **134,** 127–148 (1997).

38. Bernhardson, M. & Alexanderson, H. Early Holocene NW-W winds reconstructed from small dune fields, central Sweden. *Boreas* (2018). doi:10.1111/bor.12307

39. Bronk Ramsey, C. Recent and Planned Developments of the Program OxCal. *Radiocarbon* **55,** (2013).

40. Reimer, P. J. *et al.* IntCal13 and Marine13 Radiocarbon Age Calibration Curves 0–50,000 Years cal BP. *Radiocarbon* **55,** 1869–1887 (2013).

41. Blockley, S. P. E., Ramsey, C. B., Lane, C. S. & Lotter, A. F. Improved age

modelling approaches as exemplified by the revised chronology for the Central European varved lake Soppensee. *Quat. Sci. Rev.* **27,** 61–71 (2008).

42. Bronk Ramsey, C. Bayesian Analysis of Radiocarbon Dates. *Radiocarbon* **51,** 337–360 (2009).

43. Eldevik, T. *et al.* A brief history of climate e the northern seas from the last Glacial Maximum to global warming. *Quat. Sci. Rev.* **106,** 225–246 (2014).

44. Dokken, T. M., Nisancioglu, K. H., Li, C., Battisti, D. S. & Kissel, C. Dansgaard-Oeschger cycles: Interactions between ocean and sea ice intrinsic to the Nordic seas. *Paleoceanography* **28,** 491–502 (2013).

45. Simstich, J., Sarnthein, M. & Erlenkeuser, H. Paired d18O signals of Neogloboquadrina pachyderma (s) and Turborotalita quinqueloba show thermal stratification structure in Nordic Seas. *Mar. Micropaleontol.* **48,** 107–125 (2003).

46. Aagaard, K., Coachman, L. K. & Carmack, E. On the halocline of the Arctic Ocean. *Deep Sea Res. Part A, Oceanogr. Res. Pap.* **28,** 529–545 (1981).

47. Ezat, M. M., Rasmussen, T. L. & Groeneveld, J. Persistent intermediate water warming during cold stadials in the southeastern Nordic seas during the past 65 k.y. *Geology* **42,** 663–666 (2014).

48. Pados, T. & Spielhagen, R. F. Species distribution and depth habitat of recent planktic foraminifera in Fram Strait, Arctic Ocean. *Polar Res.* **33,** 22483 (2014).

49. Thornalley, D. J. R., Barker, S., Broecker, W. S., Elderfield, H. & McCave, I. N. The deglacial evolution of North Atlantic deep convection. *Science* **331,** 202–5 (2011).

50. Thornalley, D. J. R. *et al.* A warm and poorly ventilated deep Arctic Mediterranean during the last glacial period. *Science.* **349,** 706–710 (2015).

51. Lane, C. S., Brauer, A., Blockley, S. P. E. & Dulski, P. Volcanic ash reveals time-transgressive abrupt climate change during the Younger Dryas. *Geology* **41,** 1251–1254 (2013).

52. Jackson, R. *et al.* Asynchronous instability of the North American-Arctic and Greenland ice sheets during the last deglaciation. *Quat. Sci. Rev.* **164,** 140–153 (2017).

53. MacLeod, A., Matthews, I. P., Lowe, J. J., Palmer, A. P. & Albert, P. G. A second tephra isochron for the Younger Dryas period in northern Europe: The Abernethy Tephra. *Quat. Geochronol.* **28,** 1–11 (2015).

54. Johannsdottir, G. E., Thordarson, T. & Geirsdottir, A. The Widespread 10ka Saksunarvatn Tephra: A Product of Three Separate Eruptions? in *AGU Fall*

Meeting Abstracts (2006).

55. Barker, S. *et al.* Interhemispheric Atlantic seesaw response during the last deglaciation. *Nature* **457,** 1097–1102 (2009).

56. Shakun, J. D. *et al.* Global warming preceded by increasing carbon dioxide concentrations during the last deglaciation. *Nature* **484,** 49–54 (2012).

57. Somes, C. J. & Oschlies, A. On the influence of 'non-Redfield' dissolved organic nutrient dynamics on the spatial distribution of N<inf>2</inf> fixation and the size of the marine fixed nitrogen inventory. *Global Biogeochem. Cycles* **29,** 973–993 (2015).

58. Schmittner, A., Oschlies, A., Matthews, H. D. & Galbraith, E. D. Future changes in climate, ocean circulation, ecosystems, and biogeochemical cycling simulated for a business-as-usual CO2 emission scenario until year 4000 AD. *Global Biogeochem. Cycles* **22,** (2008).

59. Schmittner, A. *et al.* Biology and air-sea gas exchange controls on the distribution of carbon isotope ratios (δ13C) in the ocean. *Biogeosciences* **10,** 5793–5816 (2013).

60. Schmittner, A. & Galbraith, E. D. Glacial greenhouse-gas fluctuations controlled by ocean circulation changes. *Nature* **456,** 373–376 (2008).

61. Bouttes, N., Roche, D. M. & Paillard, D. Systematic study of the impact of fresh water fluxes on the glacial carbon cycle. *Clim. Past* **8,** 589–607 (2012).

62. Rahmstorf, S. *et al.* Thermohaline circulation hysteresis: a model intercomparison. *Geophys. Res. Lett.* **32,** 1–5 (2005).

63. Schmittner, A. Decline of the marine ecosystem caused by a reduction in the Atlantic overturning circulation. *Nature* **434,** 628–633 (2005).

64. Blackwell, P. G. & Buck, C. E. Estimating radiocarbon calibration curves. *Bayesian Anal.* **3,** 225–248 (2008).

65. Heaton, T. J., Blackwell, P. G. & Buck, C. E. A Bayesian approach to the estimation of radiocarbon calibration curves: The IntCal09 methodology. *Radiocarbon* **51,** 1151–1164 (2009).

66. Niu, M., Heaton, T. J., Blackwell, P. G. & Buck, C. E. The Bayesian approach to radiocarbon calibration curve estimation: the IntCal13, Marine13, and SHCal13 methodologies. *Radiocarbon* **55,** 1905–1922 (2013).
