# Supplementary material for: Deep-water circulation changes lead North Atlantic climate during deglaciation
Source: Nat Commun. 2019 Mar 20;10:1272. doi: 10.1038/s41467-019-09237-3 (PMC6426850; doi:10.1038/s41467-019-09237-3)
Supplement: Supplementary file 3 — Description of Additional Supplementary Files [file 41467_2019_9237_MOESM3_ESM.pdf]

Description of Additional Supplementary Files

- Supplementary Data 1. Major element concentrations of individual tephra shards assigned to the Abernethy Tephra.
- Supplementary Data 2. Tephra layers and planktic/benthic ^{14}C dates from core MD99-2284.
- Supplementary Data 3. Modelled Ocean-Atmosphere ^{14}C age offsets.
- Supplementary Data 4. WAIS Divide 06A Ice Core ^{10}Be data.
- Supplementary Data 5. Age offsets between IntCal13 (^{14}C) and ice cores (^{10}Be) timescales.
